# Short birth interval in the Asia-Pacific region: A systematic review and meta-analysis

**DOI:** 10.7189/jogh.14.04072

**Published:** 2024-05-03

**Authors:** Tahir Ahmed Hassen, Catherine Chojenta, Md Nuruzzaman Khan, Desalegn Markos Shifti, Melissa Leigh Harris

**Affiliations:** 1 Centre for Women’s Health Research, School of Medicine and Public Health, College of Health, Medicine and Wellbeing, University of Newcastle, Australia; 2 School of Medicine and Public Health, College of Health, Medicine and Wellbeing, University of Newcastle, Australia; 3 Department of Population Science, Jatiya Kabi Kazi Nazrul Islam University, Mymensingh, Bangladesh; 4 Child Health Research Centre, The University of Queensland, Brisbane, Australia

## Abstract

**Background:**

Short birth interval is associated with an increased risk of adverse health outcomes for mothers and children. Despite this, there is a lack of comprehensive evidence on short birth interval in the Asia-Pacific region. Thus, this study aimed to synthesise evidence related to the definition, classification, prevalence, and predictors of short birth interval in the Asia-Pacific region.

**Methods:**

Five databases (MEDLINE, Scopus, Cumulative Index to Nursing and Allied Health Literature, Maternity and Infant Care, and Web of Science) were searched for studies published between September 2000 and May 2023 (the last search was conducted for all databases in May 2023). We included original studies published in English that reported on short birth interval in the Asia-Pacific region. Studies that combined birth interval with birth order, used multi-country data and were published as conference abstracts and commentaries were excluded. Three independent reviewers screened the articles for relevancy, and two reviewers performed the data extraction and quality assessment. The risk of bias was assessed using the Joanna Briggs Institute critical appraisal tool. The findings were both qualitatively and quantitatively synthesised and presented.

**Results:**

A total of 140 studies met the inclusion criteria for this review. About 58% (n = 82) of the studies defined short birth interval, while 42% (n = 58) did not. Out of 82 studies, nearly half (n = 39) measured a birth-to-birth interval, 37 studies measured a birth-to-pregnancy, four measured a pregnancy-to-pregnancy, and two studies measured a pregnancy loss-to-conception. Approximately 39% (n = 55) and 6% (n = 8) of studies classified short birth intervals as <24 months and <33 months, respectively. Most of the included studies were cross-sectional, and about two-thirds had either medium or high risk of bias. The pooled prevalence of short birth interval was 33.8% (95% confidence interval (CI) = 23.0–44.6, *I^2^* = 99.9%, *P* < 0.01) among the studies that used the World Health Organization definition.

**Conclusions:**

This review’s findings highlighted significant variations in the definition, measurement, classification, and reported prevalence of short birth interval across the included studies. Future research is needed to harmonise the definition and classification of short birth interval to ensure consistency and comparability across studies and facilitate the development of targeted interventions and policies.

**Registration:**

PROSPERO CRD42023426975.

Inter-birth interval, which refers to the duration between two consecutive births, has received increasing attention due to its impact on fertility and the health of mothers and children [1]. The World Health Organization (WHO) recommends a minimum inter-birth interval of 33 months between two consecutive live births or a minimum interval of 24 months between birth and a subsequent pregnancy [2]. According to this definition, a birth-to-birth interval of less than 33 months or a birth-to-conception interval of less than 24 months is considered a short birth interval. The WHO’s recommendation of 24 months was based on the increased risk of adverse maternal, perinatal, and infant outcomes associated with intervals shorter than 24 months. At the time of formulating this recommendation, the 24-month interval was also chosen based on additional factors such as its alignment with the WHO and United Nations Children’s Fund recommendation of breastfeeding duration of at least two years and its simplicity to use in programs, particularly compared to 18 months or 27 months [2].

Previous studies have indicated that short birth interval is associated with adverse maternal and child health outcomes, including preterm birth [3], stillbirth [4–7], low birth weight [6,8], neonatal mortality [4,6,9,10], infant mortality [9,10], and under-five mortality [9,10]. Short birth interval is also associated with increased risk of maternal morbidity such as anaemia, high blood pressure, placental abruption, placenta previa and uterine rupture [7,11,12]. Various factors have been associated with an increased risk of experiencing short birth interval, including shorter breastfeeding duration [13,14], female sex of the previous child [13,15], death of the previous child [16], lack of use of contraception [13,14], and lack of antenatal care follow up [13]. On the other hand, factors such as residing in households with either the richer or richest wealth quintiles [14,16], higher maternal education, and younger maternal age [17] were reported to reduce the odds of experiencing short birth interval.

Although there is a lack of data on the global estimate of short birth interval, the available evidence suggests that the prevalence varies between and within countries and regions. For example, in sub-Saharan African countries, the reported prevalence of short birth interval ranges from 22–60%, with a pooled prevalence of 46.9% [18]. In Central Asia, about 33% of births were reported to have occurred within a short interval. Further, a comparative report of 72 countries using Demographic and Health Survey data has indicated that 25% of births occurred within a short interval [19]. These variations might be attributed to factors such as the definition or classifications of short birth interval used.

The Asia-Pacific region encompasses many countries with distinct sociocultural, geographical, and economic challenges that hinder the provision of high-quality maternal and child health care. Most countries in the region are of low and middle-income with higher rates of maternal and child mortality [20,21]. These poor outcomes might be partly attributed to a lack of or inadequate use of effective contraceptive methods as well as sub-optimal birth interval. For example, less than one-third of married or in-union women residing in Asia-Pacific countries such as Papua New Guinea, Pakistan, and the Solomon Islands reported using any modern contraceptive method (e.g. oral contraceptive pills). In contrast, the average prevalence of modern contraceptive use for high-income and upper-middle-income countries of the region was reported to be 62.3 and 60.4%, respectively, indicating significant variations within the region [22].

Although short birth interval has been investigated in some countries of the Asia-Pacific region [14,16,17,23–29], there are insufficient data regarding how short birth interval is defined, measured, and its prevalence and underlying factors across the entire region. Therefore, this systematic review and meta-analysis aimed to synthesise evidence related to the definition, classification, prevalence, and predictors of short birth interval in the Asia-Pacific region. Understanding these dynamics will help to inform postpartum family planning strategies and improve maternal and child health outcomes through targeted reproductive health policies and interventions in the region.

## METHODS

We conducted a systematic review and meta-analysis and reported the findings following the Preferred Reporting Items of Systematic Review and Meta-analysis (PRISMA) 2020 statement [30]. The protocol for the review was registered with PROSPERO (registration number: CRD42023426975) as part of a larger project on short birth interval in the Asia-Pacific region.

### Eligibility criteria

Human studies were eligible to be included in this systematic review if they were conducted in the Asia-Pacific region [31,32], written in English, published between September 2000 and May 2023, and reported on the epidemiology of short birth interval (including its measurement, prevalence, and predictors). Original research using qualitative, cross-sectional, case-control, cohort, quasi-experimental, non-randomised intervention, or randomised controlled trial study designs were considered. We systematically excluded studies based on specific criteria to ensure the precision and relevance of the review. First, we excluded studies if the data encompassed multiple countries, and the inability to disaggregate country-specific data for the Asia-Pacific region was evident. This exclusion aimed to maintain the focus on the Asia-Pacific context, enhancing the specificity of the analysis. Second, we excluded studies if they reported on short birth interval in conjunction with birth order without the capacity to disaggregate this information. This criterion sought to eliminate complexity and ensure a clear delineation of short birth interval patterns independent of birth order. Third, we excluded studies reporting birth interval as a continuous variable and providing mean or median values, rather than categorising the variable to define short birth interval. This criterion aimed to maintain consistency in the definition of short birth interval across studies, facilitating a more coherent synthesis of evidence. Additionally, conference abstracts, commentaries, reviews, and case reports were excluded to uphold methodological rigor and prioritise original empirical research for a more reliable and evidence-based systematic review.

### Information sources

We searched the following electronic databases: MEDLINE, Scopus, Maternity and Infant Care, Web of Science, and Cumulative Index to Nursing and Allied Health Literature. Three authors (DMS, CC, MH) developed the search strategy based on the requirements for each database. The search strategy was refined in consultation with the University of Newcastle librarian and then tested in MEDLINE and Embase. The initial search was conducted in July 2022 and updated in May 2023 for all the databases. The reference lists of published review articles were also scrutinised for additional relevant publications.

### Search strategy

Three authors (DMS, CC, MH) developed Boolean strings from key search terms and medical subject headings terms and customised them for each database. Since this systematic review was conducted as a part of a larger project which aimed to assess the epidemiology of short birth interval and its effect on maternal and child health outcomes [33], we included terms related to short birth interval, prevalence, neonatal and infant health, under-five health, and maternal health to construct search strings. We also added the list of the individual countries in the Asia-Pacific region [31,32] to the search strings, aiming to make the search hits as comprehensive and relevant as possible. The search strategy and results for each of the databases are presented in Tables S1–5 in the **Online Supplementary Document**.

### Selection process

**Figure 1:**
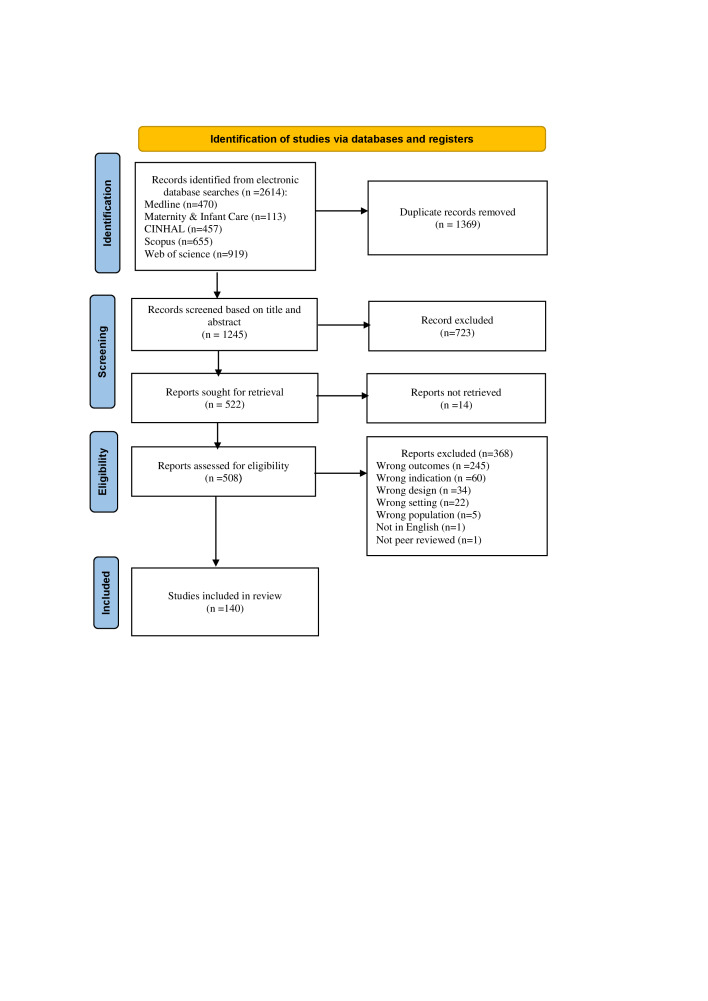
Studies’ selection process according to PRISMA 2020 Statement.

Studies were exported from each database into the EndNote, version 20.2.1 (Clarivate, Philadelphia, PA, USA) and checked for duplication. Once duplicates were removed, the studies were exported to Covidence (Veritas Health Innovation, Melbourne, Australia) for title and abstract screening and full-text review. Three authors (DMS, CC, MH) independently screened studies by title and abstracts for eligibility. If a study was deemed eligible, the whole article (full text) was independently read by three reviewers (TAH, CC, MH). Each reviewer independently assessed the study’s relevance against the pre-defined inclusion criteria. For a study in which the full text was unavailable, the authors were contacted where possible to obtain the full text. Disagreements during initial screening and full-text review were resolved through discussion between the reviewers. Any discrepancies or disagreements during this stage were thoroughly discussed among the reviewers to reach a consensus. In instances where consensus could not be reached immediately, further consultation and deliberation were undertaken with the senior members of the research team until a unanimous decision was achieved. The reasons for exclusion were documented for the studies excluded in the full-text review (**Figure 1**).

### Data collection process and data items

Data for the included studies were extracted by two independent authors (TAH, DMS) using a structured spreadsheet. Data were extracted using the following study parameters: author and year of publication, country, study setting, study design, sample size, and data collection method. Data related to the study outcome (short birth interval), including its definition or measurement, classifications, prevalence, and predictors, were also extracted for each study. For the predictors of short birth interval, the reported effect sizes and measured confounders were also extracted.

### Study risk of bias assessment

The Joanna Briggs Institute (JBI) critical appraisal tools were used to assess study quality [34,35]. The JBI critical appraisal tools assess three key aspects of the study – design, conduct, and analysis. The studies were grouped based on their designs, and the appropriate JBI tool was used for each group. Two authors (TAH, DMS) independently rated each study, and disagreements were resolved through a discussion. For each group, the total score was divided into three categories – high, medium, and low. Studies with higher scores were considered less prone to methodological bias and categorised as having a low risk of bias.

The cut-off points used to classify the total scores into three risks of bias categories were determined based on the overall scores, which varied among specific JBI tools. For instance, for cross-sectional studies, we classified studies with a total score of 7–8 as having a low risk of bias, scores of 5–6 as a medium risk of bias, and scores of 1–4 as a high risk of bias (Tables S8–9 in the **Online Supplementary Document**).

### Synthesis methods

Information on the definition or classification, measurement, prevalence, and predictors of short birth interval was summarised using narrative synthesis. Study characteristics (including author and year of publication, country, study design, and population characteristics) and key findings such as the definition, classification, terminology used, prevalence, and predictors of short birth interval and their effect measures were summarised. To estimate the pooled prevalence of short birth interval among studies adhering to the WHO recommendation, we first checked the extent of heterogeneity using *I^2^* statistics with a corresponding *P*-value. We then selected either a fixed-effect model (if the estimated *I^2^* statistic value was <75%) or a random-effect model (if the estimated *I^2^* statistic value exceeded 75%) to estimate the pooled prevalence. We explored the potential sources of heterogeneity through subgroup analyses based on the pre-defined subgroups, such as the country and the classification of short birth interval. We also checked the publication bias through visual inspection of the funnel plot and using Egger’s regression test. We also performed trim and fill analysis to adjust for missing studies. Based on the observed higher heterogeneity, we selected a random-effect model to estimate the pooled prevalence among studies that followed the WHO recommendation. For subgroup analyses, however, we used either a fixed-effect or random-effect model as appropriate. All statistical analyses were conducted using STATA, version 15.1 (Stata Corp LLC, College Station, TX, USA).

## RESULTS

### Study inclusion

A total of 2614 studies were identified from the initial search. From the total number of studies identified, 1369 duplicates were removed, and 723 were excluded during title and abstract screening. A further 368 studies were excluded after full-text review. A total of 140 studies that reported on the epidemiology of short birth interval and were published between 2000–2023 were included in this review (**Figure 1**).

### Characteristics of the included studies

Of the included studies, the majority were conducted in Bangladesh (n = 33), followed by India (n = 26) and Australia (n = 16). A large proportion of studies were either cross-sectional (n = 103) or cohort studies (n = 28). The remaining nine studies included data from case-control (n = 6) or longitudinal studies (n = 3). Further, 53 studies were specific to short birth interval and reported on either the prevalence or the predictors and 87 studies were not specific to short birth interval but considered birth interval as a covariate (Table S6 in the **Online Supplementary Document**). Characteristics of the studies that followed the WHO recommendation [14,17,36–44] for the definition and classification of short birth interval are presented in **Table 1**.

**Table 1:** Characteristics of included studies that used the World Health Organization definition of short birth interval

Author and year	Country	Study design	Classification of SBI, interval type*	Terminology used	Population characteristics	Prevalence of SBI (%)
Asif et al. 2022 [36]	Pakistan	Cross-sectional	<33, birth to birth	Birth interval	2246 women who reported their child health variable information in the PDHS	72.7
Asif et al. 2023 [37]	Pakistan	Cross-sectional	<33, birth to birth	Birth spacing	8274 women who provided complete information for all the study variables in the PDHS	69.2
Chowdhury et al. 2018 [38]	Bangladesh	Cross-sectional	<33, birth to birth	Birth interval	8588 children born singleton	17.4
Chowdhury et al. 2023 [14]	India	Cross-sectional	<33, birth to birth	Birth interval	98 522 rural mothers who had more than one child in the five years preceding the survey	51.0
DeJonge et al. 2014 [39]	Bangladesh	Cross-sectional	<33, birth to birth	Birth interval	5571 women with complete information on birth interval, pregnancy outcomes and predictors	24.6
Islam et al. 2023 [40]	Bangladesh	Cross-sectional	<33, birth to birth	Birth interval	5941 women who had at least two pregnancies	26.0
Ismail et al. 2008 [41]	Malaysia	Cross-sectional	<24, birth to pregnancy	Birth interval	355 married Malay women who delivered at least three babies	45.1
Mardiana et al. 2019 [42]	Malaysia	Cross-sectional	<24, birth to pregnancy	Inter pregnancy interval	559 antenatal mothers with two or more pregnancies	48.0
Nausheen et al. 2021 [17]	Pakistan	Cross-sectional	<33, birth to birth	Birth interval	2394 women with at least one live birth in the last six years	22.9
Qin et al. 2017 [43]	China	Retrospective cohort	<24, birth to pregnancy	Inter pregnancy interval	3309 singleton-second pregnancies	9.8
Wardani et al. 2022 [44]	Indonesia	Cross-sectional	<24, birth to pregnancy	Birth interval	3413 singleton-born infants	9.24

PDHS – Pakistan Demographic and Health Survey, SBI – short birth interval

*SBI is classified in months.

### Risk of bias assessment of the included studies

Overall, out of the 140 included studies, 113 studies demonstrated either a low risk of bias (n = 47) or medium risk of bias (n = 66), and the remaining (n = 27) had a high risk of bias. Considering the risk of bias and study designs, approximately 80% of the cross-sectional studies had either a low (n = 43) or medium (n = 48) risk of bias. The case-control studies demonstrated either a medium (n = 4) or high risk of bias (n = 2). Furthermore, 20 of the cohort studies showed either a low (n = 4) or medium (n = 16) risk of bias (Tables S7–9 in the **Online Supplementary Document**).

### Definition and classification of short birth interval

Among the 140 studies included, 39 studies considered the duration of time elapsed between the birth of the index child (child under study) and the birth of the previous child, commonly referred to as the birth-to-birth interval, to define the short birth interval. Of these 39 studies, 36 studies used the term birth interval to report short birth interval, while the remaining three studies used either inter-pregnancy interval (n = 1) [45], inter-outcome interval (n = 1) [46] or birth spacing (n = 1) [37] to describe the same concept. Additionally, 37 studies considered the duration of time between the birth of the previous child and the subsequent pregnancy, referred to as the birth-to-conception interval, and three studies [47–49] measured the time between two pregnancies, known as the pregnancy-to-pregnancy interval. Within the studies that focused on birth to conception, 34 used the term inter-pregnancy interval, while in three studies [42,50,51], the term birth interval was used. Among the studies that measured the time elapsed between two pregnancies, three studies [47,48,52] used the term birth interval, and one study [49] described this as an inter-pregnancy interval. It is also noteworthy that in approximately 40% of the studies, the specific duration used to define a short birth interval was not explicitly defined.

With the classification of short birth interval, 55 studies classified a birth interval of <24 months as a short one, of which only 12 studies measured birth-to-birth interval. Ten studies considered a birth interval of ≤24 months as a short birth interval, and of these, four studies [51,53–55] measured birth-to-birth interval, one study [47] considered the interval between two pregnancies, and the remaining five studies [56–60] did not define the short birth interval. 10 studies classified a birth interval of either <33 (n = 8) or ≤33 months (n = 2) as a short birth interval, and in all studies, the interval was measured from birth to birth. In 19 studies, an interval of <6 months (n = 18) or ≤6 months (n = 1) was classified as a short inter-pregnancy interval, of which 16 studies considered birth to pregnancy, two studies measured either medical abortion to conception (n = 1) [61] or pregnancy loss to conception intervals (n = 1) [62], and one study [63] did not define the interval. A total of 16 studies classified an interval of either <18 (n = 15) or ≤18 months (n = 1) as a short inter-pregnancy interval (n = 7) or short birth interval (n = 6). Of the studies that classified an interval of <18 or ≤18 months as a short inter-pregnancy interval, four studies [64-68] considered birth to pregnancy, whilst one study [49] measured a pregnancy-to-pregnancy interval.

In general, regarding the measured interval and the terminology used, out of 81 studies that used the term ‘birth interval’, only 35 studies measured birth-to birth-intervals and in the remaining 46 studies, the interval was either measured from pregnancy-to-pregnancy (n = 3), from birth to pregnancy (n = 3) or was not defined (n = 40). Of the 46 studies that investigated inter-pregnancy interval, only one study measured the interval between two pregnancies and the remainder either measured from birth-to-pregnancy (n = 32), medical abortion/pregnancy loss to abortion interval (n = 2), birth-to-birth (n = 1) or did not define how the interval was measured (n = 10).

### Prevalence of short birth interval

The prevalence of short birth interval varied according to the definition and classification used in the studies. In the studies that measured birth-to-birth interval and classified a birth interval of <24 or ≤24 months as a short birth interval (n = 23), the prevalence of short birth interval ranged from 5.1 [69] to 64.4% [70]. In the studies that measured birth-to-birth interval and considered a birth interval of <18 months as a short birth interval (n = 7), the reported prevalence ranged from 1.3 [71] to 42.5% [72]. For studies that measured birth-to-birth interval and considered a birth interval of <33 or ≤33 months as a short birth interval (n = 10), the reported prevalence ranged from 17.4 [38] to 72.7% [73]. When a birth-to-pregnancy interval was measured and an interval of <12 months was considered as a short birth interval (n = 5), the prevalence ranged from 4.3 [74] to 30.0% [75]. Among the studies that measured birth-to-pregnancy interval and considered an interval of <6 months as a short inter-pregnancy interval (n = 15), a prevalence of 2.9 [76] to 32.3% [77] was observed.

### Pooled prevalence of short birth interval

**Figure 2:**
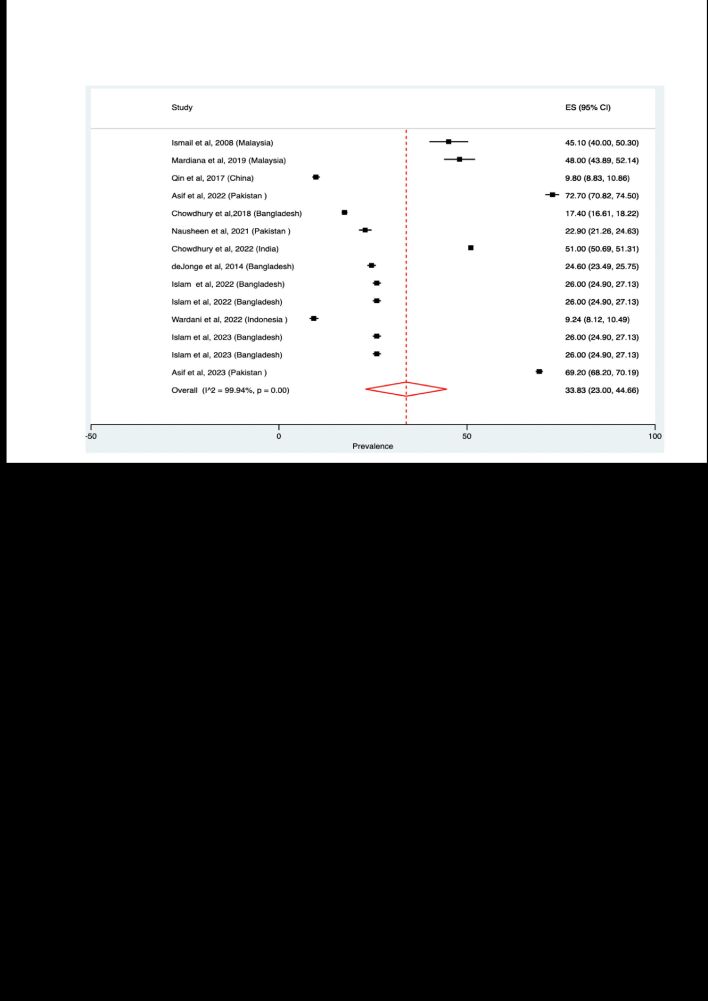
Pooled prevalence of short birth interval among studies that followed World Health Organization recommendation.

Significant variations were observed between studies included so it was not appropriate to present an overall pooled prevalence of short birth interval. However, we summarised pooled estimates based on study characteristics such as WHO recommendations to classify short birth interval, months used to classify short birth interval, and the country of the studies. Among the studies that used the WHO recommendations, the pooled prevalence was 33.8% (95% confidence interval (CI) = 23.0–44.6, *I^2^* = 99.9%, *P* < 0.01) (**Figure 2**). When months that were used to classify short birth interval was considered, the pooled prevalence ranged from 9.7% (95% CI = 8.5–10.9, *I^2^* = 99.9%, *P* < 0.01) for studies that used ≤6 months to 41.7% (95% CI = 39.7–43.6, *I^2^* = 99.9%, *P* < 0.01) for the studies that employed a ≤36-month definition. Considering the country where the studies were conducted, the pooled prevalence ranged from 7.0% (95% CI = 5.7–8.4) in Japan to 46.8% (95% CI = 43.8–52.1) in Malaysia (Table S10 and Figures S1–15 in the **Online Supplementary Document**).

Regarding the publication bias, substantial publication bias was observed based on the results of funnel plots. However, after conducting sensitivity analysis using the Trim and Fill methods (Figure S16, panel B in the **Online Supplementary Document**), the pooled estimate for the prevalence of short birth interval was found to be similar to that obtained prior to adjusting for the publication bias. This demonstrates robustness of the estimate.

### Predictors of short birth interval

Although the prevalence of short birth interval was reported in most of the included studies, the predictors or associated factors were only reported in about 9% of the studies. Sociodemographic and economic factors (e.g. place of residence, religion, maternal or husband education, maternal age, wealth index), reproductive history (e.g. use of contraception, parity), and child-related factors (e.g. gender, breastfeeding history, survival status of the previous child) were commonly investigated in relation to the short birth interval. While most studies used an adjusted odds ratio (AOR) and 95% CI, a few used other effect size measures such as prevalence rate, relative risk, and hazard ratio to report the strength of associations between the investigated factors and short birth interval. The findings highlighted that short birth interval is associated with various factors, although there were inconsistencies between the studies. Maternal education was investigated in relation to short birth interval in eight studies, of which five reported a positive association, one study reported a negative association [42] and two studies did not find a significant association [16] (Table S11 in the **Online Supplementary Document**). A study from Bangladesh reported a 1.6 increased odds of experiencing short birth interval among women with no formal education compared to those with a higher education [29]. Another community-based cross-sectional study from the same country found a much higher risk among illiterate mothers (odds ratio (OR) = 4.0; 95% CI = 1.9–17.2) [78]. A cross-sectional study from Pakistan also reported a decreased odds of the short birth interval by 25% (hazard ratio (HR) = 0.75; 95% CI = 0.7–0.9), 38% (HR = 0.6; 95% CI = 0.5–0.8), and 31% (HR = 0.69; 95% CI = 0.5–0.9) among women with a secondary, intermediate, and higher education, respectively, as compared to those with no education [17]. Conversely, a facility-based cross-sectional study from Malaysia reported increased odds of short birth interval among mothers with higher education as compared to mothers with lower education [42].

Maternal age was one of the most investigated demographic factors in relation to short birth interval. Out of the 11 studies that investigated maternal age, four reported a positive association between maternal age and short birth interval, four reported a negative association, and three found no significant association [50,54,78]. Although measured slightly differently across the studies, the findings were inconsistent, with varying risks observed across different age groups. Among the studies which measured maternal age at marriage, one community-based cross-sectional study from Bangladesh reported 25% (AOR = 1.25; 95% CI = 1.05–1.48) and 54% (AOR = 1.54; 95% CI = 1.27–1.86) increased risks of short birth interval among women aged 15–18 and >18 years, respectively, compared to those aged 10–15 years [79]. Another study from the same country, however, documented decreased odds for women aged 20–34 (prevalence ratio (PR) = 0.14; 95% CI = 0.11–0.17) and 35 years and above (PR = 0.03; 95% CI = 0.02–0.05), compared to those aged 19 years and below [16].

In a community-based cross-sectional study conducted in Pakistan, while being aged 25–30 and >30 years was found to be protective (HR = 0.63, 95% CI = 0.53–0.75; HR = 0.29, 95% CI = 0.22–0.39, respectively) of having a short birth interval, those aged 20 years and below were found to be at risk of experiencing short birth interval (HR = 1.36; 95% CI = 1.07–1.73), compared with those aged between 20–24 years [17]. Furthermore, other studies from Malaysia [42] and India [14] reported an increased risk of short birth interval among mothers aged less than 35 and 30 years of age, respectively.

Several studies also examined the association between socioeconomic status and short birth interval, mainly using wealth index or quantile (n = 4 studies), and almost all studies found an increased risk of having short birth interval among women from households with lower wealth status [14,16,29]. For instance, in a study conducted in India, the odds of experiencing short birth interval were found to be reduced by 5.0% (AOR = 0.95; 95% CI = 0.90–0.99), 9.0% (AOR = 0.91; 95% CI = 0.86–0.97) and 40% (AOR = 0.60; 95% CI = 0.55–0.66) among women from households with a poorer, middle, and richest wealth index, respectively, compared to those from households with the poorest wealth index [14]. In a community-based cross-sectional study from Bangladesh, however, only women from households with the richest wealth quantile were found to be at reduced risk of short birth interval (PR = 0.61; 95% CI = 0.45–0.85) [16].

The association between the survival status of the previous child or sibling was investigated in four studies included in this review, and in all studies, women whose previous child did not survive were found to be at increased risk of experiencing short birth interval [14,16,29,79]. For example, a Bangladeshi study and an Indian study documented five times (PR = 5.23; 95% CI = 4.18–6.55) [16] and nearly two times (AOR = 1.76; 95% CI = 1.63–1.90) [14] higher risks of experiencing short birth interval among women who had experienced the loss of their preceding child. In addition to the survival status of the previous child, factors such as family composition and desired number of sons were examined in relation to short birth interval (n = 3 studies). For example, a cross-sectional study conducted in India reported that women who desired to have fewer sons exhibited reduced odds of experiencing short birth interval. In comparison to women who had desired to have two or more sons, those who had desired to have no sons and one son had reduced odds of experiencing short birth interval by 21% (AOR = 0.79; 95% CI = 0.74–0.84) and 26% (AOR = 0.78; 95% CI = 0.75–0.82), respectively [14].

Reproductive factors such as use of contraception, duration of breastfeeding, and parity were also examined in relation to short birth interval. A shorter duration of breastfeeding or no breastfeeding of the previous child was found to increase the risk of experiencing short birth interval (n = 2 studies) [14,41], with one Indian study reporting approximately a 3-fold (AOR = 2.73; 95% CI = 2.50–2.97) increased risk for women who had not breastfed their previous child [14]. Use of contraception was found to be significantly associated with short birth interval (n = 3 studies), with increased risk among non-users [14,17,41]. The odds of experiencing short birth interval was also found to be increased with a higher number of parity (n = 2 studies) [41,42], with one Malaysian study showing a 3-fold increased risk (AOR = 3.11; 95% CI = 1.42–6.84) among mothers with a parity of more than three [42].

## DISCUSSION

This systematic review mapped evidence on short birth interval in the Asia-Pacific region by focusing on its definition, measurement, prevalence, and predictors. The findings indicate huge variations in terms of the definition of short birth interval used, the length of interval used, and the reported prevalence. Overall, most of the studies were cross-sectional and about one third had a low risk of bias or demonstrated a high quality.

The WHO suggests that the measurement of birth interval should be based on either the time between two consecutive live births or the time between birth and the next conception. In line with this, the WHO recommends a minimum interval of 33 months between two consecutive live births or a minimum interval of 24 months between a birth and the subsequent pregnancy [2]. The findings of this systematic review, however, indicated that a large proportion of studies conducted in the Asia-Pacific region did not follow this recommendation. As a result, there were inconsistencies in the definition or measurement, classification, and reported prevalence. These inconsistencies pose challenges in accurately assessing the prevalence and the impact of short birth interval on maternal and child health outcomes. Furthermore, inconsistent measurement and classification of short birth interval may lead to misinterpretation of the associated risks and suboptimal decision-making in reproductive health services, including family planning. Promoting consistency in defining and classifying short birth interval is required to accurately measure the burden of short birth interval and develop targeted interventions and policies.

The results also indicated that not only did the definition or classification of short birth interval vary across studies, but so did the terminology. These variations were also reported in previous studies [15]. Generally, in this systematic review, terminologies such as birth interval, interpregnancy interval, inter-delivery interval, inter-outcome interval, and birth spacing were identified in relation to short birth interval. In some cases, however, the terminologies used and the interval considered to measure short birth interval did not coincide, complicating the interpretation of the results. For example, of the 37 papers that used the term inter-pregnancy interval, only three studies [47,48,52] considered pregnancy-to-pregnancy interval while the rest studies used either birth-to-pregnancy or birth-to-birth interval to report on short interpregnancy or birth interval. Harmonising and using the terminologies that reflect the measured interval is also critically important to avoid misinterpretation. For example, the following terminologies and intervals may be considered. Birth interval for the interval measured between two subsequent live births (birth to birth interval), birth-to-pregnancy interval for the interval measured between birth and subsequent pregnancy, and inter-pregnancy interval for the interval measured between two consecutive pregnancies.

The observed variations in the definition or classification of short birth interval might also be attributed to a wide discrepancy in the reported prevalence rates of short birth intervals in the region, both within and between the countries. It is important to note, however, that the reported prevalence rates varied significantly even among the studies that used relatively similar approaches and were conducted in comparable settings [69,70]. This was also observed among the studies that reported following the WHO’s recommendation [14,17,36–44], indicating that not only the methodological differences but also other contextual factors might have also contributed to the observed variation.

In addition to differences in the definition and classification, the wide variations in prevalence rates can be attributed to cultural and socio-economic factors and methodological differences among studies. Cultural norms, beliefs, and traditions regarding family planning and fertility may affect the timing between births [37,80,81]. For example, certain cultures consider having a large family as a symbol of prosperity or social status, which may, in turn, contribute to short birth interval as women tend to achieve a desired family size within a short period of time [82]. In addition, some religious beliefs may also influence fertility and family planning decisions by promoting the idea of procreation, which may encourage couples to have short spacing between births [82–84].

Similarly, economic factors, such as the availability and accessibility of modern contraception and other health care services, can also influence the practice of birth interval [85,86]. Effective use of modern contraception plays an important role in pregnancy spacing and, thereby, reducing adverse maternal and child health outcomes [87–90]. Access to and use of effective contraceptive methods, however, are often negatively influenced by various economic and socio-cultural factors [91,92]. Lack of access to quality health care services, lower educational attainment, and lack of information about how to use contraceptive methods are among the highly cited factors that contribute to the inadequate use of modern contraceptives in low- and middle-income countries [93–95]. Furthermore, in some communities, traditional values may also hinder open discussions about contraception, leading to limited awareness and usage of contraceptives and sub-optimal birth spacing [96].

This systematic review also identified important factors in relation to short birth interval. Socio-demographic and economic factors, women’s reproductive history, and child-related factors were commonly investigated in relation to short birth interval. Mother's educational status showed varying associations with short birth interval across different studies, with some reporting increased risks among illiterate mothers [78] and others reporting decreased odds among women with higher education [29]. Although educated women usually tend to delay their first pregnancy [89], it has also been hypothesised that these women may want to compress motherhood into fewer years and, therefore are likely to have shorter birth intervals [97].

In most of the studies that examined the link between maternal age and short birth interval, younger maternal age was associated with higher odds of experiencing short birth interval. Younger women tend to be more fertile and sexually active and may have impaired access to and use of modern contraception, which increases the likelihood of shorter interval between pregnancies [98–100]. This review also found a positive association between socio-economic status, measured by wealth index or quantile, and short birth interval. Women from households with lower wealth or in a poorer socio-economic status had increased odds of experiencing short birth interval. This could be partly explained by the fact that wealthier women tend to have access to quality health care services, including access to modern contraception to prolong birth intervals [89].

The survival status of the previous child emerged as a prominent factor, with studies reporting higher risks of short birth interval among women who had experienced the loss of their preceding child. It has been hypothesised that women who had experienced adverse pregnancy outcomes in previous pregnancies tend to have next pregnancy soon without fully recovering from the last pregnancy [4]. Earlier literature also documented that the death of previous child is associated with premature truncation of breastfeeding, which in turn, contributes to short birth interval, particularly if modern contraception is not in place [101].

This systematic review possesses several strengths. To the best of our knowledge, this is the first review to present comprehensive evidence on short birth interval in the Asia-Pacific region. We used a comprehensive search strategy and evaluated studies published over a period of more than two decades (2000–2023). Another notable strength of this review is its inclusion and evaluation of all relevant studies incorporating birth interval as either a covariate or a main variable. This approach aimed to understand how short birth intervals were defined and classified in the Asia-Pacific region, addressing fundamental issues previously overlooked in prior reviews. Finally, the findings were reported following the updated PRISMA 2020 Statement, the recommended Statement.

This systematic review also has some limitations that need to be acknowledged. While most of the included studies explicitly mentioned the months to classify short birth interval, there were some instances where the lower categories of birth intervals were considered as short birth intervals, and this might impact the overall prevalence rate. In addition, although it was crucial to include all pertinent studies that addressed birth interval to provide a thorough understanding of its definition and classification, in some studies, particularly those that did not specifically investigate short birth interval, the definition and classification used were not explicitly reported. Although meta-analysis was conducted and the pooled prevalence was reported for the studies that followed WHO recommendations, the significant variations between the studies hindered presenting the overall pooled prevalence of short birth interval for all studies included in this review. Moreover, although we conducted subgroup analysis based on the country where studies were conducted and the classification of a short birth interval, the limited number of studies in certain sub-groups did not allow us to conduct meta-regression to quantify the effect on the overall pooled estimate.

## CONCLUSIONS

The findings of this review highlighted significant variations in the definition, measurement, classification, and reported prevalence of short birth interval across the included studies. Most importantly, we identified a misinterpretation of the WHO’s 24-month waiting period. While WHO considers two dimensions – the interval type (from live birth to subsequent pregnancy) and the duration (at least 24 months) – when formulating its recommendation, the first dimension (interval type) was overlooked, in many cases. In addition, we also identified the discordance of interval measured and the terminology used to report short birth interval. Both the misinterpretation of the WHO’s recommendation and the inappropriate use of terminologies could significantly impact the interpretation of the results. This warrants the need to harmonise the definition and classification of short birth interval, preferably by following the WHO recommendation and the need to adhere to the recommendation, to improve comparability and facilitate a better understanding of the associated risks and impacts.

Factors such as maternal education, age, and socioeconomic status showed associations with short birth interval, with younger and economically disadvantaged women at higher risk. The survival status of the previous child also played a role, with women who had experienced child death being more likely to have shorter birth intervals. Efforts should be made to promote education and access to quality health care services, including modern contraception, particularly among women with lower socio-economic status. Furthermore, understanding the impact of previous adverse pregnancy outcomes on subsequent birth interval and ensuring adequate support and care for women who have experienced such outcomes is essential to reduce the prevalence of short birth interval and improve maternal and child health outcomes.

## Additional Material


Online Supplementary Document
