# Supplementary material for: Short birth interval in the Asia-Pacific region: A systematic review and meta-analysis
Source: J Glob Health. 2024 May 3;14:04072. doi: 10.7189/jogh.14.04072 (PMC11067827; doi:10.7189/jogh.14.04072)
Supplement: Online Supplementary Document [file jogh-14-04072-s001.pdf]

Supplementary Document

Short birth interval in the Asia-Pacific region: a systematic review and meta-analysis

Table S1. Search strategy of Medline database (September 2000 to May 2023)

#	Query	Results
1	wom?n.mp. or Wom?n/	1,380,889
2	women of reproductive age.mp.	10,272
3	mother.mp. or Mothers/	176,472
4	Infant, Newborn/ or neonat*.mp.	817,400
5	Infant, Newborn/ or newborn*.mp.	833,557
6	children aged less than 28 days.mp.	0
7	Infant/ or infant*.mp.	1,391,006
8	less than one year child*.mp.	3
9	children aged less than 12 months.mp.	18
10	children less than 12 months*.mp.	75
11	under-five child*.mp.	1,688
12	under-5 child.mp.	89
13	less than five years child.mp.	0
14	Child/ or child*.mp. or Child Health/	2,745,473
15	children aged less than 59 month*.mp.	2
16	children less than 59 month*.mp.	3
17	1 or 2 or 3 or 4 or 5 or 6 or 7 or 8 or 9 or 10 or 11 or 12 or 13 or 14 or 15 or 16	4,729,513
18	"Birth Intervals"/ or "birth interval*".mp.	2,991
19	birth spacing*.mp.	1,135

20	pregnancy interval*.mp.	459
21	interpregnancy interval*.mp.	614
22	inter-pregnancy interval*.mp.	179
23	interbirth interval*.mp.	301
24	inter-birth interval*.mp.	123
25	rapid repeat pregnancy.mp.	68
26	pregnancy spacing*.mp.	92
27	repeat teenage pregnancy.mp.	10
28	child spacing*.mp.	323
29	birth to birth interval*.mp.	49
30	birth to pregnancy interval*.mp.	13
31	pregnancy to pregnancy interval*.mp.	7
32	18 or 19 or 20 or 21 or 22 or 23 or 24 or 25 or 26 or 27 or 28 or 29 or 30 or 31	4,610
33	American Samoa.mp. or American Samoa/	456
34	Afghanistan.mp. or Afghanistan/	8,179
35	Bangladesh.mp. or Bangladesh/	21,323
36	Bhutan.mp. or Bhutan/	1,255
37	Cambodia.mp. or Cambodia/	5,762
38	China.mp. or China/	380,043
39	Fiji.mp. or Fiji/	2,561
40	India.mp. or India/	181,305
41	Indonesia.mp. or Indonesia/	22,358
42	Kiribati.mp. or Micronesia/	1,423
43	Democratic People's Republic of Korea.mp. or "Democratic People's Republic of Korea"/	379
44	Lao People's Democratic Republic.mp.	665
45	Malaysia.mp. or Malaysia/	27,554
46	Maldives.mp. or Indian Ocean Islands/	1,173
47	Marshall Islands.mp. or Micronesia/	1,438

48	the Federated States of Micronesia.mp.	287
49	Mongolia.mp. or Mongolia/	6,202
50	Myanmar.mp. or Myanmar/	5,562
51	Nepal.mp. or Nepal/	15,491
52	Pakistan.mp. or Pakistan/	33,825
53	Papua New Guinea.mp. or Papua New Guinea/	5,840
54	Philippines.mp. or Philippines/	14,476
55	Samoa/ or Samoa.mp.	1,347
56	Sri Lanka.mp. or Sri Lanka/	10,336
57	Solomon Islands.mp. or Melanesia/	1,590
58	Thailand.mp. or Thailand/	43,721
59	Timor-Leste.mp. or Timor-Leste/	544
60	Tonga/ or Tonga.mp.	628
61	Tuvalu.mp. or Micronesia/	1,332
62	Vanuatu.mp. or Vanuatu/	849
63	Vietnam.mp. or Vietnam/	23,326
64	Asia-Pacific region*.mp.	2,514
65	Asia-Pacific countries.mp.	348
66	Asia-Pacific state.mp.	0
67	Australia.mp. or Australia/	194,141
68	Brunei Darussalam.mp.	280
69	French Polynesia.mp. or Polynesia/	2,537
70	Guam.mp. or Guam/	1,486
71	Hong Kong.mp. or Hong Kong/	28,137
72	Japan.mp. or Japan/	225,242
73	Republic of Korea.mp. or "Republic of Korea"/	44,002
74	Macao SAR.mp.	37
75	Nauru.mp. or Micronesia/	1,369

76	New Caledonia.mp. or New Caledonia/	1,851
77	New Zealand.mp. or New Zealand/	81,754
78	Northern Mariana Islands.mp. or Micronesia/	1,363
79	Palau.mp. or Palau/	510
80	Singapore.mp. or Singapore/	23,549
81	Singapore.mp. or Singapore/	23,549
82	Singapore.mp. or Singapore/	23,549
83	33 or 34 or 35 or 36 or 37 or 38 or 39 or 40 or 41 or 42 or 43 or 44 or 45 or 46 or 47 or 48 or 49 or 50 or 51 or 52 or 53 or 54 or 55 or 56 or 57 or 58 or 59 or 60 or 61 or 62 or 63 or 64 or 65 or 66 or 67 or 68 or 69 or 70 or 71 or 72 or 73 or 74 or 75 or 76 or 77 or 78 or 79 or 80 or 81 or 82	1,309,908
84	17 and 32 and 83	1,010
85	limit 84 to (english language and yr="2000 -Current")	470

Table S2. Search strategy of Web of Science database (September 2000 to May 2023)

#	Search	Result
1	ALL=(wom?n OR "women of reproductive age" OR mother* OR neonat* OR newborn* OR "children aged less than 28 days" OR infant* OR "less than one year child*" OR "children aged less than 12 months" OR "children less than 12 months*" OR "under-five child*" OR "under-5 child" OR "less than five years child" OR child* OR "children aged less than 59 month*" OR "children less than 59 month*")	5,428,406
2	ALL=("birth interval*" OR "birth spacing*" OR "pregnancy interval*" OR "interpregnancy interval*" OR "inter-pregnancy interval*" OR "interbirth interval*" OR "inter-birth interval*" OR "rapid repeat pregnancy" OR "pregnancy spacing*" OR "repeat teenage pregnancy" OR "child spacing*" OR "birth to birth interval*" OR "birth to pregnancy interval*" OR "pregnancy to pregnancy interval*")	3,794
3	ALL=("american samoa" OR afghanistan OR Australia OR bangladesh OR bhutan OR "Brunei Darussalam" OR cambodia OR china OR fiji OR "French Polynesia" OR Guam OR "Hong Kong" OR india OR indonesia OR Japan OR kiribati OR "Republic of Korea" OR "democratic people's republic of korea" OR "lao people's democratic republic" OR "Macao SAR" OR malaysia OR maldives OR "marshall islands" OR "the federated states of micronesia" OR mongolia OR myanmar OR Nauru OR nepal OR "New Caledonia" OR "New Zealand" OR "Northern Mariana Islands" OR pakistan OR "papua new guinea" OR palau OR philippines OR samoa OR	19,203,217

	singapore OR "sri lanka" OR "solomon islands" OR thailand OR "timor-leste" OR tonga OR tuvalu OR vanuatu OR vietnam OR "asia-pacific region*" OR "asia-pacific countries" OR "asia-pacific state")	
1&2&3	(Filtered by year (01-09-2000 to 30-05-2023) and English language	919
Total		

Table S3. Search strategy of CINAHL database (September 2000 to May 2023)

#	**Search**	**Result**
1	(wom?n OR "women of reproductive age" OR mother* OR neonat* OR newborn* OR "children aged less than 28 days" OR infant* OR "less than one year child*" OR "children aged less than 12 months" OR "children less than 12 months*" OR "under-five child*" OR "under-5 child" OR "less than five years child" OR child* OR "children aged less than 59 month*" OR "children less than 59 month*")	1,493,787
2	("birth interval*" OR "birth spacing*" OR "pregnancy interval*" OR "interpregnancy interval*" OR "inter-pregnancy interval*" OR "interbirth interval*" OR "inter-birth interval*" OR "rapid repeat pregnancy" OR "pregnancy spacing*" OR "repeat teenage pregnancy" OR "child spacing*" OR "birth to birth interval*" OR "birth to pregnancy interval*" OR "pregnancy to pregnancy interval*")	3,525
3	("american samoa" OR afghanistan OR Australia OR bangladesh OR bhutan OR "Brunei Darussalam" OR cambodia OR china OR fiji OR "French Polynesia" OR Guam OR "Hong Kong" OR india OR indonesia OR Japan OR kiribati OR "Republic of Korea" OR "democratic people's republic of korea" OR "lao people's democratic republic" OR "Macao SAR" OR malaysia OR maldives OR "marshall islands" OR "the federated states of micronesia" OR mongolia OR myanmar OR Nauru OR nepal OR "New Caledonia" OR "New Zealand" OR "Northern Mariana Islands" OR pakistan OR "papua new guinea" OR palau OR philippines OR samoa OR singapore OR "sri lanka" OR "solomon islands" OR thailand OR "timor-leste" OR tonga OR tuvalu OR vanuatu OR vietnam OR "asia-pacific region*" OR "asia-pacific countries" OR "asia-pacific state")	421,677
1&2&3	Filtered by year of publication (2000-2023) AND Filtered by language (English)	457
Total		

Table S4. Search strategy of Scopus database (September 2000 to May 2023)

#	Search	Result
1	(wom?n OR "women of reproductive age" OR mother* OR neonat* OR newborn* OR "children aged less than 28 days" OR infant* OR "less than one year child*" OR "children aged less than 12 months" OR "children less than 12 months*" OR "under-five child*" OR "under-5 child" OR "less than five years child" OR child* OR "children aged less than 59 month*" OR "children less than 59 month*")	6,468,365
2	("birth interval*" OR "birth spacing*" OR "pregnancy interval*" OR "interpregnancy interval*" OR "inter-pregnancy interval*" OR "interbirth interval*" OR "inter-birth interval*" OR "rapid repeat pregnancy" OR "pregnancy spacing*" OR "repeat teenage pregnancy" OR "child spacing*" OR "birth to birth interval*" OR "birth to pregnancy interval*" OR "pregnancy to pregnancy interval*")	5694
3	("american samoa" OR afghanistan OR Australia OR bangladesh OR bhutan OR "Brunei Darussalam" OR cambodia OR china OR fiji OR "French Polynesia" OR Guam OR "Hong Kong" OR india OR indonesia OR Japan OR kiribati OR "Republic of Korea" OR "democratic people's republic of korea" OR "lao people's democratic republic" OR "Macao SAR" OR malaysia OR maldives OR "marshall islands" OR "the federated states of micronesia" OR mongolia OR myanmar OR Nauru OR nepal OR "New Caledonia" OR "New Zealand" OR "Northern Mariana Islands" OR pakistan OR "papua new guinea" OR palau OR philippines OR samoa OR singapore OR "sri lanka" OR "solomon islands" OR thailand OR "timor-leste" OR tonga OR tuvalu OR vanuatu OR vietnam OR "asia-pacific region*" OR "asia-pacific countries" OR "asia-pacific state")	4,087,211
1&2&3	Filtered by year of publication (2000-2023) AND Filtered by language (English)	655
Total		

Table S5. Search strategy of Maternity and Infant Care database (September 2000 to May 2023)

#	Searches	Results
1	wom?n.mp. [mp=abstract, heading word, title]	121203
2	"women of reproductive age".mp. [mp=abstract, heading word, title]	1011
3	mother*.mp. [mp=abstract, heading word, title]	56170
4	neonat*.mp. [mp=abstract, heading word, title]	56595
5	newborn*.mp. [mp=abstract, heading word, title]	44440
6	"children aged less than 28 days".mp. [mp=abstract, heading word, title]	0
7	infant*.mp. [mp=abstract, heading word, title]	99941
8	"less than one year child*".mp. [mp=abstract, heading word, title]	0
9	"children aged less than 12 months".mp. [mp=abstract, heading word, title]	1
10	"children less than 12 month*".mp. [mp=abstract, heading word, title]	5
11	"under-five child*".mp. [mp=abstract, heading word, title]	74
12	"under-5 child".mp. [mp=abstract, heading word, title]	13
13	"less than five years child*".mp. [mp=abstract, heading word, title]	0

14	child*.mp. [mp=abstract, heading word, title]	72123
15	"children aged less than 59 month*".mp. [mp=abstract, heading word, title]	0
16	"children less than 59 month*".mp. [mp=abstract, heading word, title]	0
17	1 or 2 or 3 or 4 or 5 or 6 or 7 or 8 or 9 or 10 or 11 or 12 or 13 or 14 or 15 or 16	236863
18	"birth interval*".mp. [mp=abstract, heading word, title]	420
19	"birth spac*".mp. [mp=abstract, heading word, title]	194
20	"pregnancy interv*".mp. [mp=abstract, heading word, title]	287
21	"interpregnancy interv*".mp. [mp=abstract, heading word, title]	328
22	"inter-pregnancy interv*".mp. [mp=abstract, heading word, title]	83
23	"interbirth interv*".mp. [mp=abstract, heading word, title]	20
24	"inter-birth interv*".mp. [mp=abstract, heading word, title]	17
25	"rapid repeat pregnan*".mp. [mp=abstract, heading word, title]	37
26	"pregnancy spac*".mp. [mp=abstract, heading word, title]	33
27	"repeat teenage pregnan*".mp. [mp=abstract, heading word, title]	7
28	"child spac*".mp. [mp=abstract, heading word, title]	57
29	"birth to birth interv*".mp. [mp=abstract, heading word, title]	11
30	"birth to pregnancy interv*".mp. [mp=abstract, heading word, title]	6
31	"pregnancy to pregnancy interv*".mp. [mp=abstract, heading word, title]	3
32	18 or 19 or 20 or 21 or 22 or 23 or 24 or 25 or 26 or 27 or 28 or 29 or 30 or 31	1146
	American Samoa.mp. [mp=abstract, heading word, title]	6
	Afghanistan.mp. [mp=abstract, heading word, title]	226
	Bangladesh.mp. [mp=abstract, heading word, title]	999

	Bhutan.mp. [mp=abstract, heading word, title]	33
	Cambodia.mp. [mp=abstract, heading word, title]	161
	China.mp. [mp=abstract, heading word, title]	2658
	Fiji.mp. [mp=abstract, heading word, title]	38
	India.mp. [mp=abstract, heading word, title]	2565
	Indonesia.mp. [mp=abstract, heading word, title]	514
	Kiribati.mp. [mp=abstract, heading word, title]	8
	Democratic People's Republic of Korea.mp. [mp=abstract, heading word, title]	1
	Lao People's Democratic Republic.mp. [mp=abstract, heading word, title]	37
	"Marshall Islands".mp. [mp=abstract, heading word, title]	7
	"the Federated States of Micronesia".mp. [mp=abstract, heading word, title]	2
	"Mongolia".mp. [mp=abstract, heading word, title]	47
	Myanmar.mp. [mp=abstract, heading word, title]	126
	Nepal.mp. [mp=abstract, heading word, title]	719
	Pakistan.mp. [mp=abstract, heading word, title]	779
	"Papua New Guinea".mp. [mp=abstract, heading word, title]	139
	Philippines.mp. [mp=abstract, heading word, title]	323
	Samoa.mp. [mp=abstract, heading word, title]	25
	"Sri Lanka".mp. [mp=abstract, heading word, title]	213
	"Solomon Islands".mp. [mp=abstract, heading word, title]	16
	Thailand.mp. [mp=abstract, heading word, title]	566
	"Timor-Leste".mp. [mp=abstract, heading word, title]	29

	Tonga.mp. [mp=abstract, heading word, title]	8
	Tuvalu.mp. [mp=abstract, heading word, title]	3
	Vietnam.mp. [mp=abstract, heading word, title]	356
	"Asia-Pacific region*".mp. [mp=abstract, heading word, title]	36
	"Asia-Pacific countr*".mp. [mp=abstract, heading word, title]	3
	"Asia-Pacific stat*".mp. [mp=abstract, heading word, title]	0
	33 or 34 or 35 or 36 or 37 or 38 or 39 or 40 or 41 or 42 or 43 or 44 or 45 or 46 or 47 or 48 or 49 or 50 or 51 or 52 or 53 or 54 or 55 or 56 or 57 or 58 or 59 or 60 or 61 or 62 or 63	9417
	17 and 32 and 64	100
	limit 65 to yr="2000 -Current"	113

Table S6: Summary of studies included to investigate the epidemiology of short birth interval in the Asia-Pacific region, grouped by classification of shorth birth interval

Author and year	Country	Study design	Classification of SBI & Interval type	Terminology used	Population characteristics	Prevalence of SBI /short IPI(%)
Panaretto et al 2006 [1]	Australia	Prospective cohort	<12: Birth to pregnancy	Interpregnancy interval	456 singleton births	30
Yamashita et al 2015 [2]	Japan	Retrospective cohort	<12: Birth to pregnancy	Interpregnancy interval	547 women with previous preterm birth	12.9
Yang et al 2019 [3]	Australia	Cross-sectional	<12: Birth to pregnancy	Interpregnancy interval	467 pregnant women following a live birth	20.9
Zhang et al 2018 [4]	China	Cross-sectional	<12: Birth to pregnancy	Interpregnancy interval	9,663 women with live births	8.9
Lin et al 2020 [5]	China	Retrospective cohort	<12: Birth to pregnancy	Interpregnancy interval	9,552 women who had delivered their second child	4.3
Negi et al 2006 [6]	Not mentioned	Prospective cohort	<12: Not defined	Interpregnancy interval	172 pregnant women	33.7
Abbas et al 2015 [7]	Pakistan	Cross-sectional	<12: Not defined	Not mentioned	220 mothers with newly born babies	22
Khan et al 2020 [8]	Bangladesh	Cross-sectional	<18: Birth to birth	Birth interval	6,340 children aged 0-59 months	1.3
Mozumder et al 2000 [9]	Bangladesh	Cross-sectional	<18: Birth to birth	Birth interval	1,887 children aged 6-39 months	8.7
Abedin et al 2020 [10]	Bangladesh	Cross-sectional	<18: Birth to birth	Birth interval	12,391 ever-married women	20.2
Abedin et al.2020 [11]	Bangladesh	Cross-sectional	<18: Birth to birth	Birth interval	327 ever-married women	42.5
Ahmad et al 2020 [12]	Indonesia	Cross-sectional	<18: Birth to birth	Birth interval	14,720 births to women of childbearing age	3.1

Holowko et al 2018 [13]	Australia	Longitudinal	<18: Birth to pregnancy	birth to pregnancy interval	6,899 women who responded to the baseline survey and Survey seven of the ALSWH*	43
Dhamrait et al 2022 [14]	Australia	Retrospective cohort	<18: Birth to pregnancy	Interpregnancy interval	32,324 singleton children	43
Nakamura et al 2022 [15]	Japan	Retrospective cohort	<18: Birth to pregnancy	Interpregnancy interval	592 births that occurred after caesarean delivery	30
Mishra et al 2022 [16]	Australia	Prospective cohort	<18: Birth to birth	Birth interval	6,130 women who were born in 1973-1978 and had two or more births	40
Kaur et al 2014 [17]	India	Cross-sectional	<18: Pregnancy to pregnancy	Interpregnancy interval	271 mothers who were admitted to hospital with a gestational age of >28 weeks and delivered singleton baby	26.4
Kluckow et al 2018 [18]	Solomon Islands	Cross-sectional	<18: Birth to pregnancy	Interpregnancy interval	1,441 women who had attended antenatal care	29.7
Kumar et al 2015 [19]	India	Cross-sectional	<18: Not defined	Birth interval	149 women who had attended antenatal care	40
Williams et al 2008 [20]	India	Cross-sectional	<18: Not defined	Birth interval	80,164 births (excluding first order birth) occurred to women of reproductive age	15.4
Kader et al 2014 [21]	India	Cross-sectional	<18: Not defined	Interpregnancy interval	20,946 infants born to women who gave birth at least once during 5 years preceding the survey	10.4
Hazel et al 2022 [22]	Nepal	Cross-sectional	<18: Not defined	Interpregnancy interval	31,424 singletons with size-for-gestational status	36
Lin et al 2022 [23]	China	Retrospective cohort	<23.5: Birth to pregnancy	Interpregnancy interval	5,951 women with singleton pregnancies whose both first and second births at the IPMCH	43
Khan et al 2021 [24]	Bangladesh	Cross-sectional	<24: Birth to birth	Birth interval	60,44 under-five children	50.4
Naz et al 2021 [25]	Pakistan	Cross-sectional	<24: Birth to birth	Birth interval	19,190 under-five children	67.6
Rahman et al 2016 [26]	Bangladesh	Cross-sectional	<24: Birth to birth	Birth interval	7,530 singleton born under-five children	19.3
Singh et al 2021 [27]	Nepal	Cross-sectional	<24: Birth to birth	Birth interval	131 women aged between 18 and 49 years, married, and who had at least two deliveries	51.1
Wali et al 2021 [28]	Multiple countries*	Cross-sectional	<24: Birth to birth	Birth interval	564518 children aged 0-59 months	15.5
Abeywickrama et al2020 [29]	Sri Lanka	Cross-sectional	<24: Birth to birth	Birth interval	7,713 live and singleton births records to ever-married women	5.1
Alam et al 2007 [30]	Bangladesh	Cross-sectional	<24: Birth to birth	Birth interval	179,292 livebirths	15.1
Amir-ud-Din et al2021 [31]	Multiple countries (32)	Cross-sectional	<24: Birth to birth	Birth interval	1,467,728 women	36.6

Bhowmik et al2017 [32]	Bangladesh	Cross-sectional	<24: Birth to birth	Birth interval	7,647 under-five children	7.6
Habib et al 2018 [33]	Pakistan	Cross-sectional	<24: Birth to birth	Birth interval	7,491 non-pregnant women	58.7
Akter et al2010 [34]	Bangladesh	Cross-sectional	<24: Birth to birth	Birth interval	10,145 ever-married women who had given birth at least to one child	19.1
Kamal et al 2022 [35]	Bangladesh	Cross-sectional	<24: Birth to birth	Birth interval	16,100 children of second and higher-order births	12
Hale et al2009 [36]	Bangladesh	Cross-sectional	<24: Birth to birth	Interactome interval	93,521 singleton live births between	13.5
Murtaza et al 2022 [37]	Pakistan	Cross-sectional	<24: Birth to birth	Interpregnancy interval	2,798 women with singleton pregnancies	20
Ismail et al 2008 [38]	Malaysia	Cross-sectional	<24: Birth to pregnancy	Birth interval	355 married Malay women who delivered their third babies and above women	45.1
Wardani et al 2022 [39]	Indonesia	Cross-sectional	<24: Birth to pregnancy	Birth interval	3,413 singleton born infants	9.24
Qin et al 2017 [40]	China	Retrospective cohort	<24: Birth to pregnancy	Interpregnancy interval	3,309 singleton second pregnancies	9.8
Mardiana et al 2019 [41]	Malaysia	Cross-sectional	<24: Birth to pregnancy	Interpregnancy interval	559 antenatal mothers with second or more pregnancy	48
Kibria et al 2018 [42]	Afghanistan	Cross-sectional	<24: Pregnancy to pregnancy	Birth interval	19,636 children/neonates	24.8
DasGupta et al 2019 [43]	Afghanistan	Cross-sectional	<24: Pregnancy to pregnancy	Birth interval	2,773 children born in healthy facilities	18.7
Omari et al 2021 [44]	Indonesia	Case-control	<24: Not defined	Interpregnancy interval	64 women who were admitted with obstetric haemorrhage during 2015–2019	6.25
Kiik et al 2021 [45]	Indonesia	Cross-sectional	<24: Not defined	Birth interval	154 ex-refuge women who had children aged 24-59 months	55.8
Meitei et al 2022 [46]	India	Cross-sectional	<24: Not defined	Birth interval	233,763 children born to women aged 15 to 49 years, in the 5 years preceding the survey	16
Myo et al 2021 [47]	Myanmar	Cross-sectional	<24: Not defined	Birth interval	220 mothers under 6 months postpartum	68.2
Pal et al 2021 [48]	Afghanistan	Cross-sectional	<24: Not defined	Birth interval	1,640 ever-married women	67.1
Patel et al 2021 [49]	India	Cross-sectional	<24: Not defined	Birth interval	120,507 children aged 6-59 months	31.1
Pravana et al 2017 [50]	Nepal	Casee-control	<24: Not defined	Birth interval	292 children aged 6-59	43.5

Rachmawati et al 2022 [51]	Indonesia	Cross-sectional	<24: Not defined	Birth interval	10, 014 women who had given birth in the five years preceding the survey	8
Rahman et al 2022 [52]	Bangladesh	Cross-sectional	<24: Not defined	Birth interval	7,886 ever-married women aged between 15 and 49 years	4.5
Rayhan et al 2006 [53]	Bangladesh	Cross-sectional	<24: Not defined	Birth interval	5,419 under-five children	11.3
Sanjel et al 2019 [54]	Nepal	Cross-sectional	<24: Not defined	Birth interval	362 women who delivered live baby in the last 12 months	52.7
Shakya et al 2001 [55]	Nepal	Cross-sectional	<24: Not defined	Birth interval	4,351 singleton births	39.6
Shukla et al 2020 [56]	India	Cross-sectional	<24: Not defined	Birth interval	77,914 children born of birth order 2 and above in the five years preceding the survey	42
Singh et al 2021b [57]	India	Cross-sectional	<24: Not defined	Birth interval	6,482 children	44.6
Singh et al 2013 [58]	India	Cross-sectional	<24: Not defined	Birth interval	30,782 children born of birth order 2 and above-five years preceding the survey	36.3
Sultana et al 2019 [59]	Bangladesh	Cross-sectional	<24: Not defined	Birth interval	6,965 children aged 0-59 months	6.8
Talukder 2017 [60]	Bangladesh	Cross-sectional	<24: Not defined	Birth interval	7,102 children aged 0-59 months	64.4
Talukder et al 2018 [61]	Bangladesh	Cross-sectional	<24: Not defined	Birth interval	6,965 children aged 0-59 months	63.9
Warrohmah et al 2018 [62]	Indonesia	Cross-sectional	<24: Not defined	Birth interval	14,727 singletons live-born infants	7.8
Ashok et al2015 [63]	India	Cross-sectional	<24: Not defined	Birth interval	600 children aged 1-5 years	27.3
Dahal et al 2021 [64]	Nepal	Case-control	<24: Not defined	Birth interval	150 under-five children	12
Das et al 2020 [65]	India	Cross-sectional	<24: Not defined	Birth interval	3,578 singleton children aged 0-23 months	18
Farid-ul-Hasnain et al 2010 [66]	Pakistan	Cross-sectional	<24: Not defined	Birth interval	800 children (non-biological children or children with visible congenital anomalies were excluded)	40.4
Hakim et al 2021 [67]	Pakistan	Cross-sectional	<24: Not defined	Birth interval	500 multiparous pregnant women	59.9
Helova et al 2017 [68]	Pakistan	Cross-sectional	<24: Not defined	Birth interval	7,399 births in the last 5 years preceding the survey	24.4

Hosain et al 2006 [69]	Bangladesh	Longitudinal	<24: Not defined	Birth interval	350 pregnant women	18.9
Chungkham et al2020 [70]	India	Cross-sectional	<24: Not defined	Birth interval	159,862 children (excluding first-born)	26.7
Sanin et al 2023 [71]	Bangladesh	Cross-sectional	<24: Not defined	Birth interval	7,562 under-five children	6.8
Das et al 2022 [72]	India	Cross-sectional	<24: Not defined	Birth interval	145,270 mothers-children's pairs	9.4
Wulandari et al 2022 [73]	Indonesia	Cross-sectional	<24: Not defined	Birth interval	14,918 women aged 15–49 years with infants born within five years before 2017	6.5
Acharya et al 2022 [74]	Nepal	Cross-sectional	<24: Not defined	Birth spacing	305 mothers who had children under the age of 6 months	23
Wang et al 2018 [75]	China	Longitudinal	<24: Not defined	Interpregnancy interval	128 women who delivered two sequential live singleton births and were diagnosed with GDM during their first pregnancies	17.2
Bhagwan et al2016 [76]	India	Cross-sectional	<24: Not defined	Interpregnancy interval	136 recently delivered mothers (between one and half to five months postpartum)	5.8
Rana et al 2015 [77]	Nepal	Cross-sectional	<24: Not defined	Not mentioned	246 children aged 0-59 months	13.4
Angeles-Agdeppa et al2019 [78]	Philippines	Longitudinal	<24: Not defined	Not mentioned	290 children aged 0-23 months in 2003	32.4
Rana et al 2021 [79]	India	Cross-sectional	<25: Not defined	Birth interval	201,874 singleton children aged 6-59 months	16.6
Haq et al 2022 [80]	Bangladesh	Cross-sectional	<25: Not defined	Birth interval	51,791 women of reproductive age (15-49 years)	35.4
Pathak et al2004 [81]	India	Cross-sectional	<30: Not defined	Interpregnancy interval	283 pregnant women	41
Asif et al 2022 [82]	Pakistan	Cross-sectional	<33: Birth to birth	Birth interval	2,246 women who reported their child health variable information in the PDHS	72.7
Chowdhury et al2018 [83]	Bangladesh	Cross-sectional	<33: Birth to birth	Birth interval	8,588 children born singleton	17.4
Nausheen et al 2021 [84]	Pakistan	Cross-sectional	<33: Birth to birth	Birth interval	2,394 women with at least 1 live birth in the last 6 years	22.9
Chowdhury et al 2022 [85]	India	Cross-sectional	<33: Birth to birth	Birth interval	98,522 rural mothers who had more than 1 child in 5 years preceding the survey	51

deJonge et al 2014 [86]	Bangladesh	Cross-sectional	<33: Birth to birth	Birth interval	5,571 women with complete information on birth interval, pregnancy outcomes, and predictors	24.6
Islam et al 2023 [87]	Bangladesh	Cross-sectional	<33: Birth to birth	Birth interval	5,941 women who had at least two pregnancies	26
Islam et al 2023b [88]	Bangladesh	Cross-sectional	<33: Birth to birth	Birth interval	5,941 mother-child dyads where women had at least two pregnancies	26
Asif et al 2023 [89]	Pakistan	Cross-sectional	<33: Birth to birth	Birth spacing	8,274 women who provided complete information for all the study variables in the PDHS	69.2
Kashem et al 2020 [90]	Bangladesh	Cross-sectional	<36: Birth to birth	Birth interval	128 rural women aged between 15 and 49 years and who had at least two live births	21.9
Tuz-zahura et al 2022 [91]	Bangladesh	Cross-sectional	<36: Birth to birth	Birth interval	5,441 births recorded to women of reproductive age group	23
Sk et al 2021 [92]	India	Cross-sectional	<36: Not defined	Birth interval	731 mothers/caregivers with at least one child aged between 36 and 59 months	23.1
Thakur et al 2018 [93]	Nepal	Cross-sectional	<36: Not defined	Birth spacing	192 children who had visited paediatrics OPD with their mother and parents	42.1
Atamou et al 2023 [94]	Indonesia	Cross-sectional	<36: Not defined	Birth spacing	166 mothers with children aged 24-59 months	19.3
Huo et al 2013 [95]	China	Prospective cohort	<6: Abortion to conception	Interpregnancy interval	4,682 nulliparous women with one mifepristone-induced abortion in their first pregnancy	21.5
Marinovich 2021 [96]	multiple countries**	Retrospective cohort	<6: Birth to pregnancy	Interpregnancy interval	3,213,855 singleton births	Not reporte d
Regan et al 2019 [97]	Australia	Retrospective cohort	<6: Birth to pregnancy	Interpregnancy interval	174,200 first and second births	3.0
Regan et al 2019b [98]	Australia	Retrospective cohort	<6: Birth to pregnancy	Interpregnancy interval	3,521 women with previous stillbirths	23
Shi et al 2021 [99]	China	Cross-sectional	<6: Birth to pregnancy	Interpregnancy interval	13,231 non primi women with live births	10.4
Tanigawa et al 2021 [100]	Japan	Retrospective cohort	<6: Birth to pregnancy	Interpregnancy interval	55,203 singleton live birth pregnancies	2.9
Tessema et al 2021[101]**	Australia	Prospective cohort	<6: Birth to pregnancy	Interpregnancy interval	306,639 women with singleton births and previous births	7.4
Xu et al 2022 [102]	China	Cross-sectional	<6: Birth to pregnancy	Interpregnancy interval	725,392 first and second-born sibling pairs of multiparous mothers	6.8
Zafar et al 2020 [103]	Pakistan	Cross-sectional	<6: Birth to pregnancy	Interpregnancy interval	285 women with singleton pregnancies	32.3

Zhang et al 2018b [104]	China	Retrospective cohort	<6: Birth to pregnancy	Interpregnancy interval	227,352 women with their singleton first and second delivery	4.1
Arshad et al 2021 [105]	Pakistan	Prospective cohort	<6: Birth to pregnancy	Interpregnancy interval	420 women with singleton pregnancies and gestational age of >28 weeks	Not reported
Ball et al 2014 [106]	Australia	Retrospective cohort	<6: Birth to pregnancy	Interpregnancy interval	40,441 mothers who had their first three births as a liveborn singleton	6.6
DaVanzo et al 2007 [107]	Bangladesh	Cross-sectional	<6: Birth to pregnancy	Interpregnancy interval	66,759 pregnancies including those that resulted in multiple births.	5.6
Dhamrait et al2021 [108]	Australia	Retrospective cohort	<6: Birth to pregnancy	Interpregnancy interval	34,574 singletons born in Western Australia with a 2009, 2012, or 2015 Australian Early Development Census (AEDC) record.	4.9
Kannaujiya et al 2020 [109]	India	Cross-sectional	<6: Birth to pregnancy	Interpregnancy interval	52,825 most recent births	12.1
Tanigawa et al 2023 [110]	Japan	Retrospective cohort	<6: Birth to pregnancy	Interpregnancy interval	55,203 singleton live-birth pregnancies	2.9
Razzaque et al2005 [111]	Bangladesh	Cross-sectional	<6: Birth to pregnancy	Interpregnancy interval	11,122 women who visited a health center during their third pregnancy between 1996 and 2002	Not reported
Hussain et al 2002 [112]	Pakistan	Cross-sectional	<6: Not defined	Birth interval	4,488 singleton live births born to 912 ever married	17.7
Reddy et al 2022 [113]	India	Case-control	≤12: Not defined	Interpregnancy interval	383 Preterm and term neonates (case vs control)	5.7
Ashfaq et al 2017 [114]	Pakistan	Cross-sectional	≤18: Birth to pregnancy	Interpregnancy interval	150 Females who gave birth to preterm	78
Khan et al 2019 [115]	Bangladesh	Cross-sectional	≤24: Birth to birth	Birth interval	7,884 children who were born in the last 5 years preceding the survey and a complete set of data for selected variables	8
Hercus et al 2020 [116]	Australia	Retrospective cohort	≤24: Birth to birth	Birth interval	2,003 multigravida women who gave birth in the selected hospitals	43.9
Sayem et al 2011 [117]	Bangladesh	Cross-sectional	≤24: Birth to birth	Birth interval	524 women with at least one birth	64.4
Karkee et al 2016 [118]	Nepal	Cross-sectional	≤24: Birth to birth	Birth interval	338 pregnant multiparous women	23
Rahman et al 2021 [119]	Bangladesh	Cross-sectional	≤24: Pregnancy to pregnancy	Birth interval	26,145 children	23.9
Sahu et al 2015 [120]	India	Cross-sectional	≤24: Not defined	Birth interval	4,112 under-five children	45.3

Sharma et al 2020 [121]	India	Cross-sectional	≤24: Not defined	Birth interval	2,105 children aged 6-23 months	34.5
Basit et al 2012 [122]	India	Case-control	≤24: Not defined	Birth interval	162 children aged 1-5 years	44.6
Shakeel et al 2022 [123]	Pakistan	Cross-sectional	≤24: Not defined	Birth interval	12,262 women aged 15-49	38.9
Singal et al 2018 [124]	India	Case-control	≤24: Not defined	Birth spacing	400 women with moderate to severe anemia (n=200) and without anemia (n=200)	28.5
Islam et al 2022 [125]	Bangladesh	Cross-sectional	≤33: Birth to birth	Birth interval	5,941 women who had at least two pregnancies of which the most recent one occurred in the five years of the survey and ended with live birth	26
Islam et al 2022b [126]	Bangladesh	Cross-sectional	≤33: Birth to birth	Birth interval	5,941 women who had at least two pregnancies of which the most recent one occurred in the five years of the survey and ended with live birth	26
Viramgami et al 2019 [127]	India	Cross-sectional	≤36: Birth to birth	Birth interval	480 working and nonworking women of reproductive age group	83.6
HarshaKumar et al 2014 [128]	India	Cross-sectional	≤36: Not defined	Interpregnancy interval	988 women who gave birth in selected hospitals	18.9
Roberts et al 2016 [129]	Australia	Retrospective cohort	≤6: Pregnancy loss to conception	Interpregnancy interval	4,290 women who conceived within 2 years	Not reporte d
Bauserman et al2020 [130]	Pakistan	Prospective cohort	6-17: Birth to birth	Inter delivery interval	29,457 women with an index pregnancy and with Inter delivery interval of 6-180 months	19.8
Khan et al 2016 [131]	Bangladesh	Cross-sectional	Not mentioned: Birth to birth	Birth interval	11,832 ever-married women who had at least one birth in the preceding five years of the survey	Not reporte d
Upadhyay et al 2005 [132]	Philippines	Prospective cohort	Not mentioned: Birth to pregnancy	Birth interval	11,23 married and fecund women	Not reporte d
Mehata et al 2014 [133]	Nepal	Cross-sectional	Not mentioned: Birth to pregnancy	Interpregnancy interval	5391 women who had a child in the last five years	28
Gebremedhin et al 2021 [134]	Australia	Retrospective cohort	Not mentioned: Birth to pregnancy	Interpregnancy interval	358046 women who delivered 3 or more consecutive singleton births	Not reporte d
Gebremedhin et al 2019 [135]	Australia	Retrospective cohort	Not mentioned: Birth to pregnancy	Interpregnancy interval	358,046 women who delivered 3 or more consecutive singleton births at 20-44 weeks	Not reporte d

Gebremedhin et al 2021b [136]	Australia	Prospective cohort	Not mentioned: Birth to pregnancy	Interpregnancy interval	mothers who had their first two (n=252368) and three consecutive (n=96315) singleton birth	Not reported
Gebremedhin et al 2021c [137]	Australia	Prospective cohort	Not mentioned: Birth to pregnancy	Interpregnancy interval	169,896 mothers who had their first two consecutive births	Not reported
Murphy et al 2001 [138]	China	Cross-sectional	Not mentioned: Not defined	Birth interval	32,172 singleton births	Not reported
Shaikh et al 2022 [139]	Pakistan	Cross-sectional	Not mentioned: Not defined	Interpregnancy interval	145 women with a history of miscarriage and singleton births	Not reported
Fatima et al 2021 [140]	Pakistan	Cross-sectional	Not mentioned: Not defined	Interpregnancy interval	190 women with singleton uncomplicated pregnancies	not reported

ALSWH: Australian Longitudinal Study on Women's Health; GDM: Gestational diabetes mellitus; PDHS: Pakistan Demographic and Health Survey: IPI: Inter-pregnancy interval; Data were disaggregated from an international study*

Risk of bias assessment of the included studies

Table S7: Joanna Briggs Institute risk of bias assessment for **Cross-sectional studies**

S.no	Authors/year	Item1	Item2	Item3	Item4	Item5	Item6	Item7	Item8	Total	Risk of bias
**1.**	Kashem et al 2020	Y	Y	Y	Y	Y	Y	Y	Y	8	Low
**2.**	Kaur et al 2014	N	Y	Y	N	Y	Y	Y	Y	6	Medium
**3.**	Khan et al 2016	Y	Y	Y	N	Y	Y	N	Y	7	Low

**4.**	Khan et al 2021	N	Y	N	N	Y	Y	N	Y	4	High
**5.**	Khan et al 2019	Y	Y	N	N	Y	Y	N	Y	5	Medium
**6.**	Khan et al 2020	Y	Y	Y	Y	Y	Y	Y	Y	8	Low
**7.**	Kibria et al 2018	Y	Y	Y	Y	Y	Y	Y	Y	8	Low
**8.**	Kiik et al 2021	Y	Y	Y	Y	Y	Y	N	Y	7	Low
**9.**	Kluckow et al 2018	N	Y	Y	N	Y	Y	N	Y	6	Medium
**10.**	Kumar et al 2015	Y	Y	Y	N	Y	Y	N	Y	6	Medium
**11.**	Mardiana et al 2019	Y	Y	Y	Y	Y	Y	Y	Y	8	Low
**12.**	Mehata et al 2014	N	Y	N	Y	Y	Y	N	Y	5	Medium
**13.**	Meitei et al 2022	Y	Y	Y	N	Y	Y	N	Y	6	Medium
**14.**	Mozumder et al 2000	Y	Y	Y	Y	Y	Y	Y	Y	8	Low
**15.**	Murphy et al 2001	Y	Y	Y	N	Y	Y	Y	Y	7	Low
**16.**	Myo et al 2021	Y	Y	Y	Y	Y	Y	Y	Y	8	Low
**17.**	Nausheen et al 2021	Y	Y	Y	Y	Y	Y	Y	Y	8	Low
**18.**	Naz et al 2021	Y	Y	N	N	Y	Y	Y	Y	6	Medium
**19.**	Pal et al 2021	N	N	Y	N	Y	Y	N	Y	4	High
**20.**	Pathak et al2004	Y	Y	N	N	Y	Y	Y	Y	6	Medium
**21.**	Pravana et al 2017	Y	Y	N	N	Y	Y	Y	Y	6	Medium
**22.**	Rachmawati et al 2022	N	Y	N	N	Y	Y	Y	Y	7	low
**23.**	Rahman et al 2021	Y	Y	Y	Y	Y	Y	Y	Y	8	low
**24.**	Rahman et al 2016	Y	Y	Y	Y	Y	Y	Y	Y	8	low
**25.**	Rahman et al 2022	Y	Y	N	N	Y	Y	Y	Y	6	Medium
**26.**	Rana et al 2021	Y	Y	N	N	Y	Y	N	Y	5	Medium
**27.**	Rana et al 2015	Y	Y	N	N	Y	Y	Y	Y	6	Medium
**28.**	Rayhan et al 2006	Y	Y	N	N	Y	Y	Y	Y	6	Medium
**29.**	Razzaque et al2005	Y	Y	Y	Y	Y	Y	N	Y	7	Low
**30.**	Sahu et al 2015	Y	Y	N	N	Y	Y	N	Y	5	Medium
**31.**	Sanajaoba et al 2010	Y	Y	N	N	Y	Y	N	Y	5	Medium
**32.**	Sanjel et al 2019	Y	Y	N	N	Y	Y	Y	Y	6	Medium
**33.**	Sayem et al 2011	N	N	N	N	Y	Y	Y	Y	4	High
**34.**	Shaikh et al 2022	Y	Y	N	N	Y	Y	N	Y	5	Medium
**35.**	Shakya et al 2001	Y	Y	N	N	Y	Y	Y	Y	6	Medium
**36.**	Sharma et al 2020	N	Y	N	N	Y	Y	Y	Y	5	Medium
**37.**	Shi et al 2021	Y	Y	Y	Y	Y	Y	Y	Y	8	Low
**38.**	Shukla et al 2020	Y	Y	N	Y	Y	Y	Y	Y	7	Low

**39.**	Singh et al 2021	Y	Y	N	N	Y	Y	Y	Y	6	Medium
**40.**	Singh et al 2013	Y	Y	N	N	Y	Y	Y	Y	6	Medium
**41.**	Singh et al 2021	N	Y	Y	Y	Y	Y	Y	Y	7	Low
**42.**	Sk et al 2021	Y	Y	N	N	Y	Y	N	Y	5	Medium
**43.**	Sultana et al 2019	Y	Y	N	N	Y	Y	N	Y	6	Medium
**44.**	Talukder 2017	Y	Y	N	N	Y	Y	N	Y	5	Medium
**45.**	Talukder et al 2018	N	Y	N	N	Y	Y	N	Y	4	High
**46.**	Thakur et al 2018	Y	Y	N	N	Y	Y	N	Y	5	Medium
**47.**	Tuz-zahura et al 2022	Y	Y	Y	Y	Y	Y	Y	Y	8	Low
**48.**	Viramgami et al 2019	N	N	N	N	Y	Y	Y	Y	4	High
**49.**	Wali et al 2021	Y	Y	Y	Y	Y	Y	Y	Y	8	Low
**50.**	Warrohmah et al 2018	Y	N	N	N	Y	Y	N	Y	4	High
**51.**	Williams et al 2008	Y	Y	Y	Y	Y	Y	Y	Y	8	Low
**52.**	Xu et al 2022	Y	Y	Y	Y	Y	Y	Y	Y	8	Low
**53.**	Yang et al 2019	Y	Y	Y	Y	Y	Y	Y	Y	8	Low
**54.**	Zafar et al 2020	Y	Y	N	N	Y	Y	Y	Y	6	Medium
**55.**	Zhang et al 2018	Y	Y	Y	Y	Y	Y	Y	Y	8	Low
**56.**	Abbas et al 2015	N	Y	N	N	Y	Y	N	Y	4	High
**57.**	Abedin et al 2020	Y	Y	N	N	Y	Y	Y	Y	6	Medium
**58.**	Abedin et al.2020	Y	Y	N	N	Y	Y	Y	Y	6	Medium
**59.**	Abeywickrama et al2020	Y	Y	Y	Y	Y	Y	Y	Y	8	Low
**60.**	Ahmad et al 2020	Y	Y	Y	Y	Y	Y	Y	Y	8	Low
**61.**	Akter et al2010	Y	Y	Y	Y	Y	Y	Y	Y	8	Low
**62.**	Alam et al2017	N	N	N	N	Y	Y	Y	Y	4	High
**63.**	Amir-ud-Din et al2021	Y	Y	Y	Y	Y	Y	Y	Y	8	Low
**64.**	Ashfaq et al 2017	N	Y	N	N	Y	Y	N	N	3	High
**65.**	Ashok et al2015	Y	Y	N	U	Y	Y	Y	U	5	Medium
**66.**	Asif et al 2022	Y	Y	Y	Y	Y	U	Y	Y	7	Low
**67.**	Bhagwan et al2016	Y	N	Y	Y	Y	U	Y	U	5	Medium
**68.**	Bhowmik et al2017	Y	Y	U	U	Y	Y	Y	Y	6	Medium
**69.**	Chowdhury et al2018	Y	Y	Y	Y	Y	Y	Y	Y	8	Low
**70.**	Chowdhury et al 2022	Y	Y	Y	Y	Y	Y	Y	Y	8	Low

**71.**	Chungkham et al2020	Y	Y	Y	Y	Y	Y	Y	Y	8	Low
**72.**	DasGupta et al 2019	N	Y	N	Y	Y	Y	Y	Y	6	Medium
**73.**	Das et al 2020	Y	U	U	N	Y	Y	Y	Y	5	Medium
**74.**	DaVanzo et al 2007	Y	Y	Y	Y	Y	Y	Y	Y	8	Low
**75.**	deJonge et al 2014	Y	Y	Y	N	Y	N	Y	Y	6	Medium
**76.**	Farid-ul-Hasnain et al 2010	Y	N	N	N	Y	Y	Y	Y	5	Medium
**77.**	Fatima et al 2021	Y	Y	N	N	N	N	N	Y	3	High
**78.**	Habib et al 2018	Y	Y	N	N	N	N	N	Y	3	High
**79.**	Hakim et al 2021	N	Y	U	U	Y	Y	Y	Y	5	Medium
**80.**	Hale et al2009	Y	Y	N	N	Y	Y	Y	Y	6	Medium
**81.**	Haq et al 2022	Y	Y	N	N	Y	Y	U	Y	5	Medium
**82.**	HarshaKumar et al 2014	Y	Y	N	N	Y	Y	Y	Y	6	Medium
**83.**	Helova et al 2017	N	Y	U	U	Y	Y	Y	Y	5	Medium
**84.**	Hussain et al 2022	U	Y	N	N	Y	Y	Y	Y	5	Medium
**85.**	Islam et al 2022	Y	Y	Y	Y	Y	Y	Y	Y	8	Low
**86.**	Islam et al 2022	Y	Y	Y	Y	Y	Y	Y	Y	8	Low
**87.**	Ismail et al 2008	Y	Y	Y	Y	Y	Y	Y	Y	8	Low
**88.**	Kader et al 2014	Y	Y	N	Y	Y	Y	Y	Y	7	low
**89.**	Kamal et al 2022	Y	Y	Y	Y	Y	Y	Y	Y	8	Low
**90.**	Kannaujiya et al 2020	Y	Y	Y	Y	Y	Y	Y	Y	8	Low
**91.**	Karkee et al 2016	Y	Y	Y	Y	Y	Y	Y	Y	8	Low
**92.**	Hazel et al 2022	Y	Y	N	N	Y	Y	Y	Y	6	Medium
**93.**	Murtaza et al 2022	Y	Y	Y	Y	Y	Y	Y	Y	8	Low
**94.**	Wardani et al 2022	N	Y	N	N	Y	Y	Y	Y	5	Medium
**95.**	Sanin et al 2023	Y	N	N	N	Y	Y	Y	Y	5	Medium
**96.**	Das et al 2022	Y	Y	N	N	Y	Y	Y	Y	6	Medium
**97.**	Wulandari et al 2022	Y	Y	N	N	Y	Y	Y	Y	6	Medium
**98.**	Acharya et al 2022	N	N	N	N	Y	Y	Y	Y	4	High
**99.**	Islam et al 2023	Y	Y	Y	Y	Y	Y	Y	Y	8	Low
**100.**	Islam et al 2023	Y	Y	Y	Y	Y	Y	Y	Y	8	Low
**101.**	Asif et al 2023	Y	Y	Y	Y	Y	Y	Y	Y	8	Low
**102.**	Atamou et al 2023	Y	N	N	N	Y	Y	Y	Y	5	Medium

**103.**	Shakeel et al 2023	Y	Y	N	N	Y	Y	Y	Y	6	Medium

Items and coding

Item1–Were the criteria for inclusion in the sample clearly defined?
Item2–Were the study subjects and the setting described in detail?
Item3– Was the exposure measured in a valid and reliable way?
Item4– Were objective, standard criteria used for measurement of the condition?
Item5– Were confounding factors identified?
Item6– Were strategies to deal with confounding factors stated?
Item7– Were the outcomes measured in a valid and reliable way?
Item8– Was there appropriate statistical analysis?
Coding: Y=Yes, N=No, U=Unclear
Note: *Low risk of bias (7-8); medium risk of bias (5-6), high risk of bias (1-4)*

Table S8: Joanna Briggs Institute risk of bias assessment for **cohort studies**

	**Authors/year**	**Item 1**	**Item 2**	**Item 3**	**Item 4**	**Item 5**	**Item 6**	**Item 7**	**Item8**	**Item9**	**Item 10**	**Item 11**	**Total**	**Risk of bias**
1.	Lin et al 2020	U	U	Y	Y	Y	U	Y	Y	U	U	N	5	High
2.	Marinovich 2021	U	U	Y	Y	Y	U	Y	Y	U	U	N	5	High
3.	Mishra et al 2022	Y	Y	Y	Y	Y	U	Y	Y	Y	Y	Y	10	Low
4.	Negi et al 2006	U	U	Y	Y	Y	U	Y	Y	U	U	N	5	High
5.	Panaretto et al 2006	Y	Y	Y	Y	Y	U	Y	Y	Y	Y	Y	7	Medium
6.	Qin et al 2017	U	U	Y	Y	Y	Y	Y	U	U	U	Y	6	Medium
7.	Regan et al 2019	Y	Y	Y	Y	Y	Y	Y	U	U	Y	Y	9	Low
8.	Regan et al 2019	Y	Y	Y	Y	Y	Y	Y	U	U	Y	Y	9	Low
9.	Roberts et al 2016	U	U	Y	Y	Y	Y	Y	U	U	Y	Y	8	Medium
10.	Tanigawa et al 2021	N	N	Y	Y	Y	Y	Y	U	U	U	Y	7	Medium
11.	Tessema et al 2021	Y	Y	Y	Y	Y	Y	Y	U	U	U	Y	8	Medium
12.	Upadhyay et al 2005	U	N	N	Y	Y	Y	Y	U	U	Y	Y	6	Medium
13.	Yamashita et al 2015	N	N	Y	Y	Y	Y	Y	U	U	U	Y	6	Medium
14.	Zhang et al 2018	Y	Y	Y	Y	Y	U	Y	U	U	N	Y	8	Medium
15.	Arshad et al 2021	N	N	N	N	N	U	Y	N	N	Y	Y	3	High
16.	Ball et al 2014	Y	Y	Y	Y	Y	Y	Y	U	U	U	Y	8	Medium
17.	Bauserman et al2020	U	U	N	Y	Y	Y	Y	U	U	U	Y	5	High
18.	Dhamrait et al2021	Y	Y	Y	Y	Y	Y	Y	U	U	U	Y	8	Medium
19.	Dhamrait et al 2022	Y	Y	Y	Y	Y	Y	Y	U	U	U	Y	8	Medium
20.	Gebremedhin et al 2021	Y	Y	N	Y	Y	Y	Y	U	U	U	Y	7	Medium
21.	Gebremedhin et al 2019	Y	Y	N	Y	Y	Y	Y	U	U	U	Y	7	Medium
22.	Gebremedhin et al 2021	Y	Y	N	Y	Y	Y	Y	U	U	U	Y	7	Medium
23.	Gebremedhin et al 2021	Y	Y	N	Y	Y	Y	Y	U	U	U	Y	7	Medium
24.	Hercus et al 2020	U	U	N	Y	Y	Y	Y	U	U	U	Y	5	High
25.	Huo et al 2013	U	U	N	Y	Y	Y	Y	U	U	U	Y	5	High
26.	Nakamura et al 2022	Y	Y	Y	Y	Y	U	Y	Y	Y	Y	Y	10	Low
27.	Lin et al 2022	U	N	N	Y	Y	Y	Y	N	U	U	Y	5	High
28.	Tanigawa et al 2023	Y	Y	Y	Y	Y	U	Y	Y	U	U	Y	10	Medium

Items and coding

Item1– Were the two groups similar and recruited from the same population?
Item2– Were the exposures measured similarly to assign people to both exposed and unexposed groups?
Item3– Was the exposure measured in a valid and reliable way?
Item4– Were confounding factors identified?
Item5– Were strategies to deal with confounding factors stated?
Item6– Were the groups/participants free of the outcome at the start of the study (or now of exposure)?
Item7– Were the outcomes measured in a valid and reliable way?
Item8– Was the follow up time reported and sufficient to be long enough for outcomes to occur?
Item9– Was follow up complete, and if not, were the reasons to loss to follow up described and explored?
Item10–Were strategies to address incomplete follow up utilized?
Item11–Was appropriate statistical analysis used?
Coding: Y=Yes, N=No, U=Unclear
Note: *Low risk of bias (9-11); medium risk of bias (6-8), high risk of bias (1-5)*

Table S9: Joanna Briggs Institute risk of bias assessment for **case-control studies**

S.No	**Authors/year**	**Item 1**	**Item 2**	**Item 3**	**Item 4**	**Item 5**	**Item 6**	**Item 7**	**Item8**	**Item9**	**Item 10**	**Total**	**Risk of bias**
1.	Omari et al 2021	Y	Y	U	N	U	Y	Y	Y	U	Y	6	Medium
2.	Rizwan et al 2020	N	N	N	N	U	Y	Y	N	U	N	2	High
3.	Singal et al 2018	N	N	N	N	U	Y	Y	U	N	N	2	High
4.	Basit et al 2012	Y	Y	U	N	U	Y	Y	Y	U	Y	6	Medium
5.	Dahal et al 2021	Y	Y	N	N	N	Y	Y	Y	N	Y	6	Medium
6.	Reddy et al 2022	Y	Y	N	N	N	Y	Y	Y	U	Y	6	Medium

Items and coding

Item1– Were the groups comparable other than the presence of disease in cases or the absence of disease in controls?
Item2– Were cases and controls matched appropriately?
Item3– Were the same criteria used for identification of cases and controls?
Item4– Was exposure measured in a standard, valid and reliable way?
Item5– Was exposure measured in the same way for cases and controls?
Item6– Were confounding factors identified?
Item7– Were strategies to deal with confounding factors stated?
Item8– Were outcomes assessed in a standard, valid and reliable way for cases and controls?
Item9– Was the exposure period of interest long enough to be meaningful?
Item10– Was appropriate statistical analysis used?
Coding: Y=Yes, N=No, U=Unclear
Note: *Low risk of bias (8-10); medium risk of bias (5-7), high risk of bias (1-4)*

Table S10: Pooled prevalence of short birth interval by months used to classify short birth interval and countries in which the studies were conducted.

Characteristics	Number of studies	Pooled prevalence (95%CI)
**Months used to classify short birth interval**		
<6	14	9.72 (8.50-10.93)
<12	5	15.78 (11.71-19.85)
<18	15	25.86 (18.81-32.90)
<24	55	28.33 (24.72-31.73)
≤24	12	40.25 (30.58-49.92)
<25	2	19.13 (18.98-19.28)
<33	8	38.72 (25.06-52.39)
≤33	2	26.0 (25.21-26.79)
<36	6	24.07 (21.70-26.44)
≤36	2	41.67 (39.71-43.64)
**Country of the studies**		
Afghanistan	3	36.84 (16.74-56.93)
Australia	11	24.05 (20.06-28.04)
Bangladesh	33	25.40 (21.84-28.95)
China	9	13.61 (11.63-15.60)
India	29	26.78 (22.57-31.00)
Indonesia	9	13.75 (10.15-17.34)
Japan	4	7.07 (5.71-8.44)
Malaysia	2	46.87 (43.89-52.14)
Nepal	10	33.38 (28.63-38.12)
Pakistan	15	42.94 (30.94-54.94)
Multiple countries	2	28.05 (27.99-28.11)

Table S11: Factors investigated in relation to short birth interval among studies included for the review.

Author and year	Country	Factors																										
		Maternal age	Mothers' education	Husband's education	Marital status	Husband's income	Women's 3E*	Wealth index or quintile	Household asset	Employment status	Type of family	Sex of the household head	Religion	Social group	Birthplace	Region of residence	Mass media exposure	Contraception	Parity	Breastfeeding	Desired number of sons	Survival status of child*	Total number of children	Birth order of preceding child	Average distance to HF	Scores of availabilities of	General health service	Decision making authority
Kashem et al 2020	Bangladesh	o	+	u	o	u	u	u	u	o	u	u	o	u	u	u	u	u	u	u	u	u	u	u	u	u	u	u
Khan et al 2016	Bangladesh	-	+	u	u	u	u	-	u	u	u	u	u	u	+	u	u	u	-	u	u	-	u	u	u	u	u	u
Mardiana et al 2019	Malaysia	+	+	u	u	u	u	u	u	u	u	u	u	u	u	u	u	u	+	u	u	u	u	u	u	u	u	u
Nausheen et al 2021*	Pakistan	+	-	u	u	u	u	u	u	u	u	u	u	u	u	u	u	+	u	u	u	u	u	u	u	u	u	u
Sayem et al 2011**	Bangladesh	o	u	u	u	o	u	u	u	u	o	u	u	u	+	u	-	u	u	u	u	u	o	u	u	u	u	o
Tuz-Zahura et al 2022	Bangladesh	+	u	o	u	u	-	u	u	-	u	u	+	u	o	+	o	u	u	u	u	-	u	o	u	u	u	u
Chowdhury et al 2022	India	-	+	u	u	+	u	-	u	u	u	u	u	+	u	-	o	+	u	+	-	+	u	u	u	u	u	u

Author and year	Country	Factors																										
		Maternal age	Mothers' education	Husband's education	Marital status	Husband's income	Women's 3E*	Wealth index or quintile	Household asset	Employment status	Type of family	Sex of the household head	Religion	Social group	Birthplace	Region of residence	Mass media exposure	Contraception	Parity	Breastfeeding	Desired number of sons	Survival status of child*	Total number of children	Birth order of preceding child	Average distance to HF	Scores of availabilities of	General health service	Decision making authority
DeJonge et al 2014	Bangladesh	+	+	u	u	u	u	u	u	u	u	u	-	u	u	u	u	u	u	u	u	u	u	u	u	u	u	u
Islam et al 2022	Bangladesh	-	o	o	u	u	u	-	u	o	u	-	u	u	u	o	o	u	u	u	u	+	o	u	+	-	o	u
Ismail et al 2008	Malaysia	-	u	u	u	u	u	u	u	u	u	u	u	u	u	u	u	+	+	+	u	u	u	u	u	u	u	u
Murtaza et al 2022	Pakistan	o	o	o	u	u	u	o	u	o	u	u	u	u	u	o	u	u	u	u	u	u	u	u	u	u	u	u

Key: + = positive association; - = negative association; O = no association; u = unreported

*3E (empowerment, education, economic status) the total mean score was derived from five indicators of empowerment and one indicator for both education and economic status-and the coverage was classified into four categories: s below 50%, 50% - 75%, 75% - 100%, and full coverage in 3E (100%). Under full coverage of women's 3E, a woman is fully empowered, educated, and economically prosperous. **Survival status of previous child, HF: Health facility

Supplementary Figures

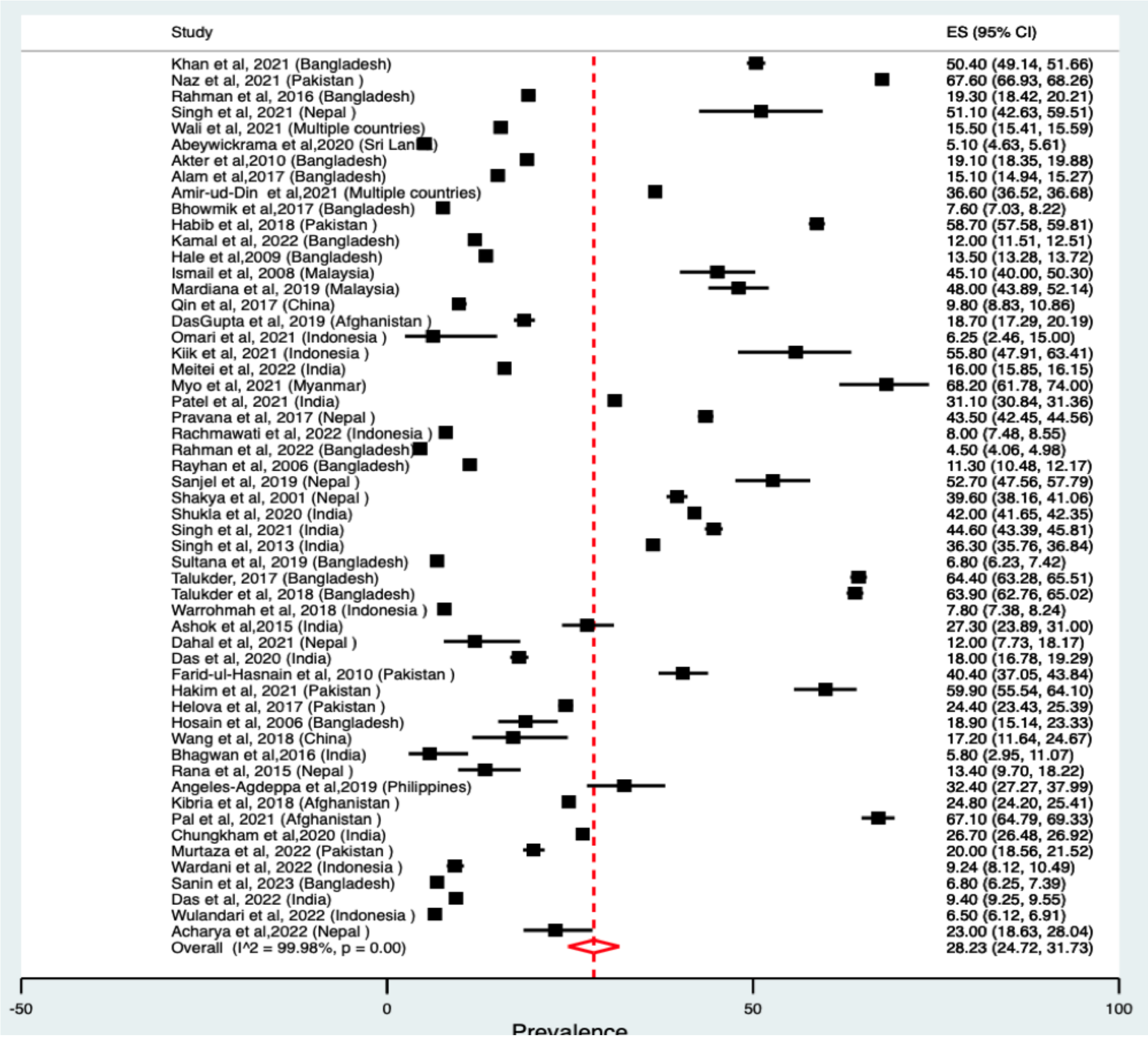

Supplementary Figure 1: Summary prevalence of short birth interval among studies an interval of < 24 months to classify shorth birth interval

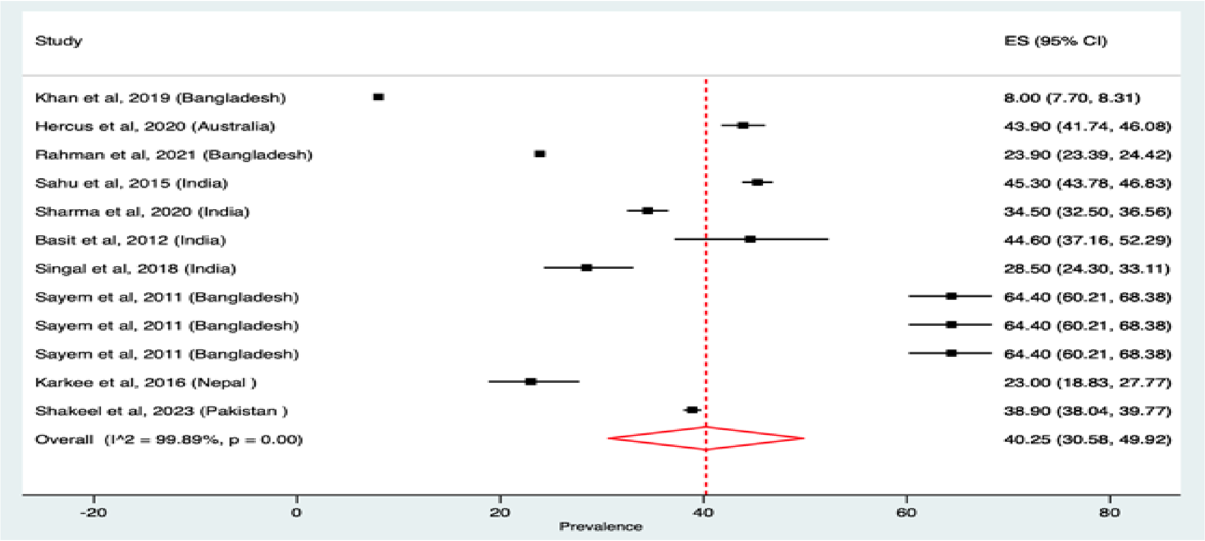

Supplementary Figure 2: Summary prevalence of short birth interval among studies an interval of ≤ 24 months to classify shorth birth interval

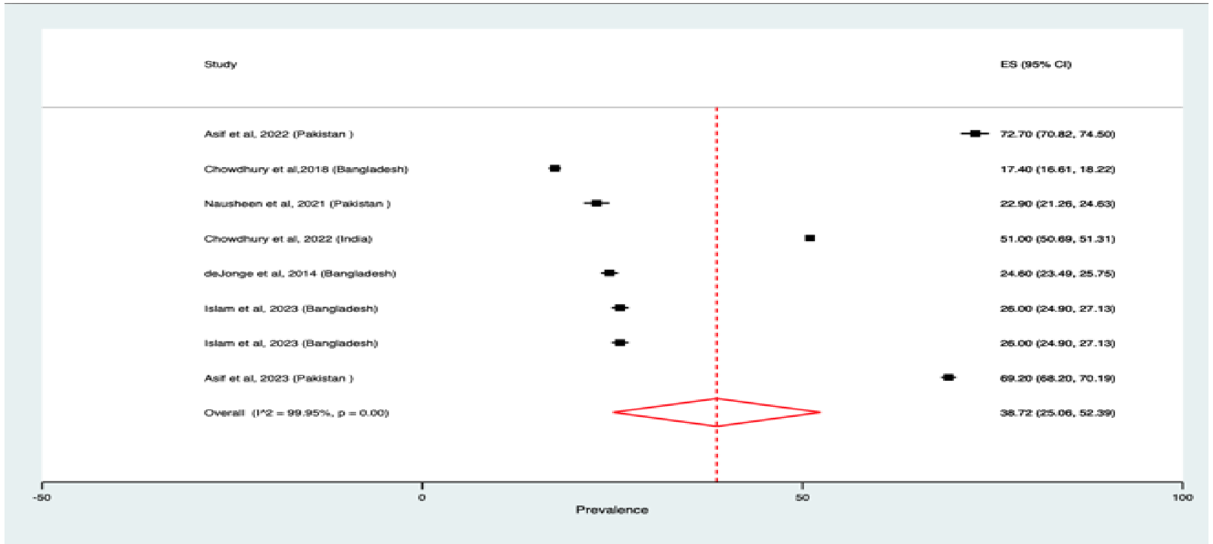

Supplementary Figure 3: Summary prevalence of short birth interval among studies an interval of < 33 months to classify shorth birth interval

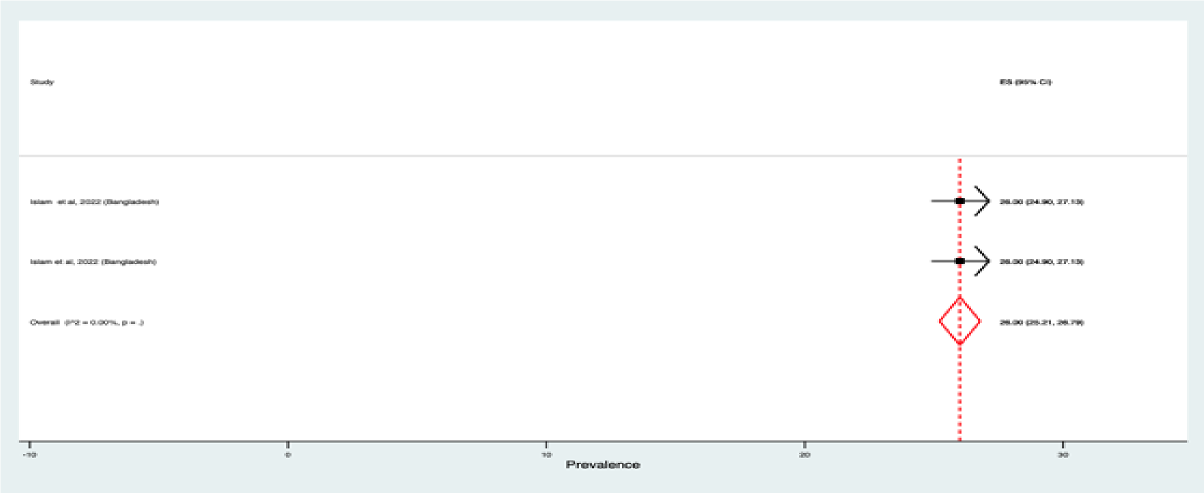

Supplementary Figure 4: Summary prevalence of short birth interval among studies an interval of ≤33 months to classify shorth birth interval

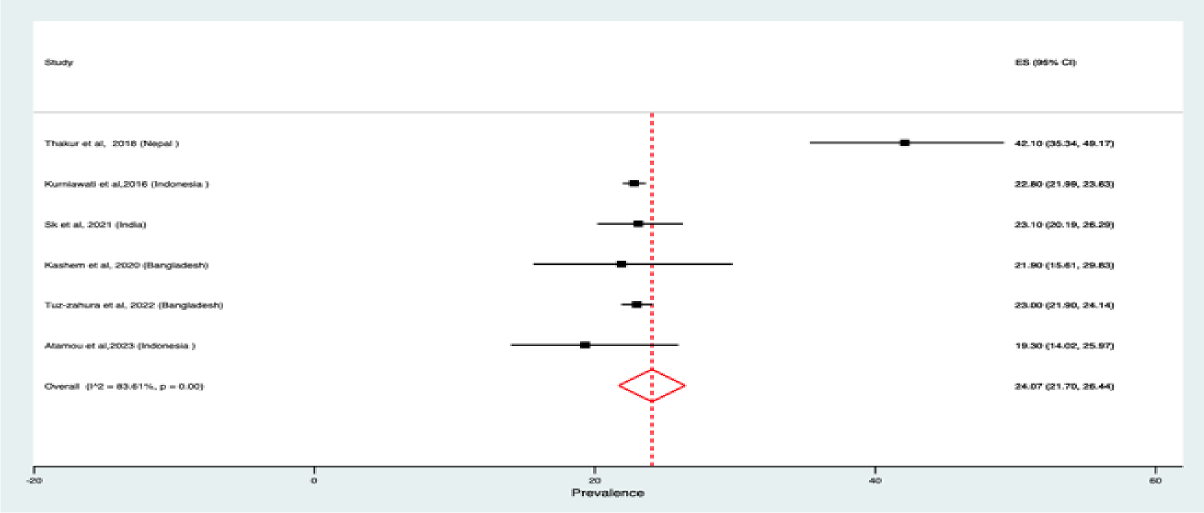

Supplementary Figure 5: Summary prevalence of short birth interval among studies an interval of< 36 months to classify shorth birth interval

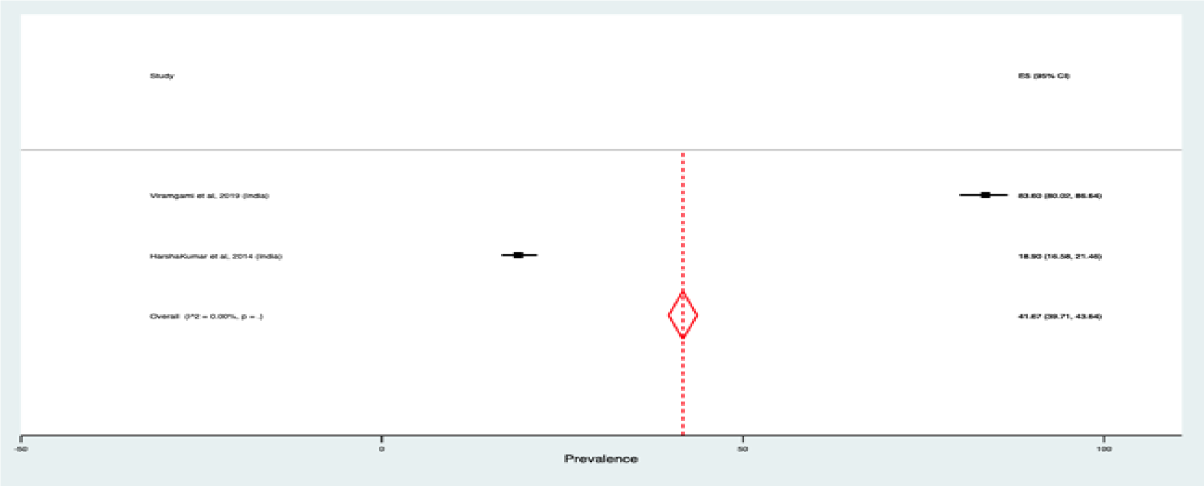

Supplementary Figure 6: Summary prevalence of short birth interval among studies an interval of ≤36 months to classify shorth birth interval

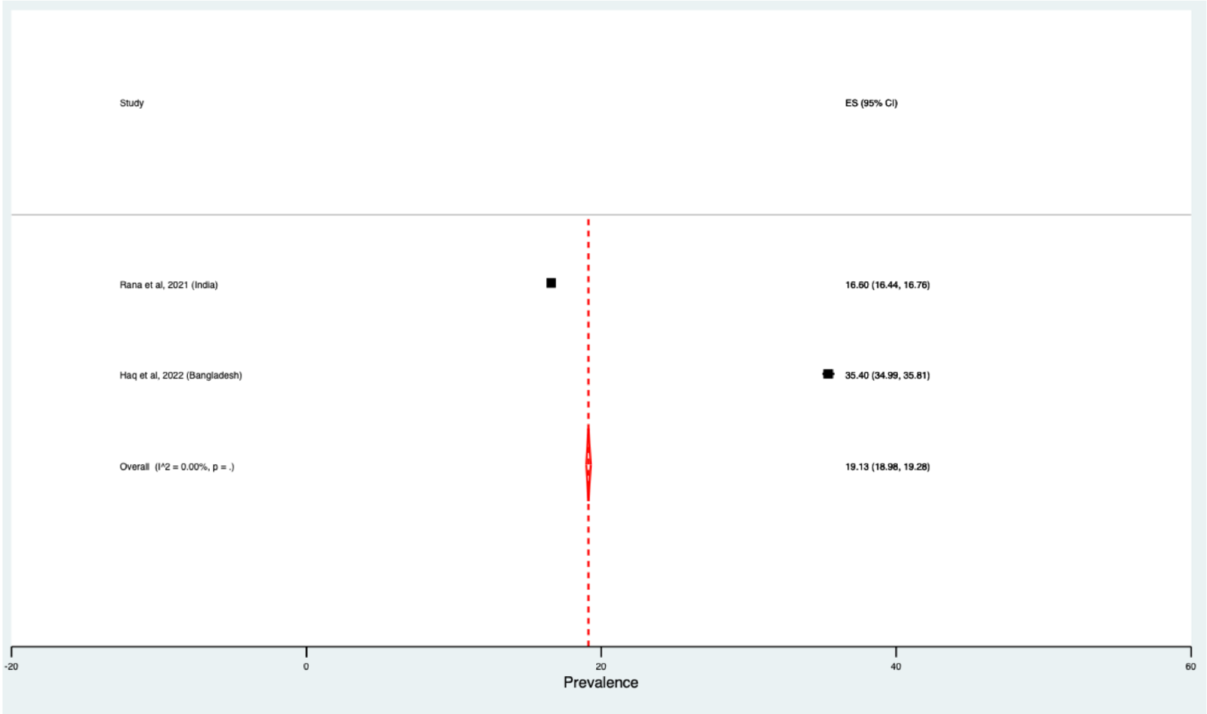

Supplementary Figure 7: Summary prevalence of short birth interval among studies an interval of < 25 months to classify shorth birth interval

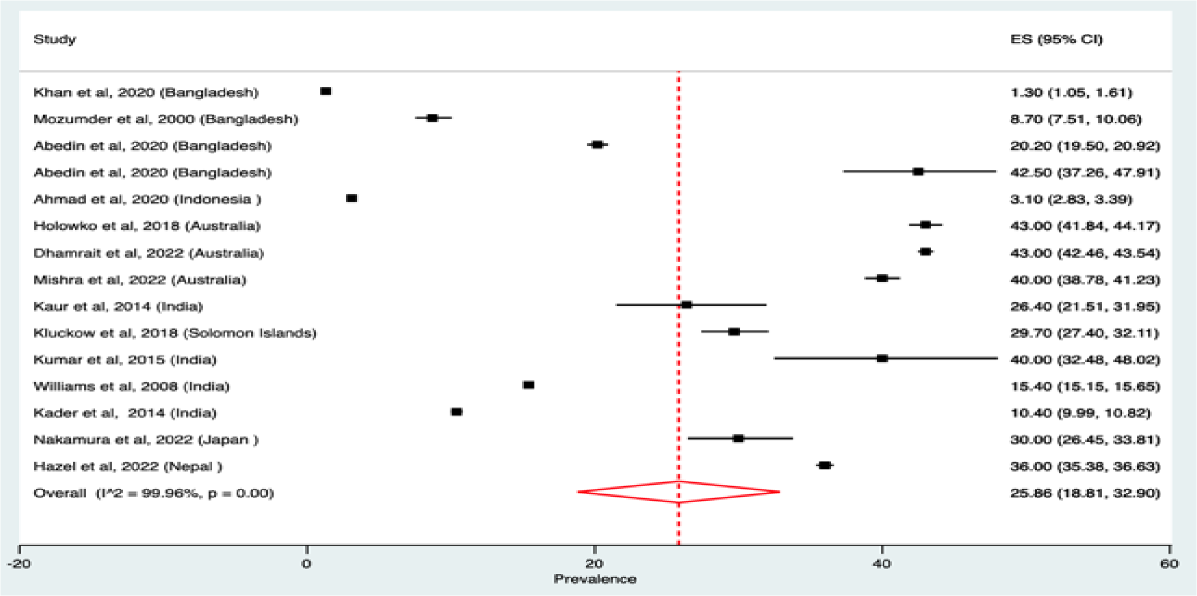

Supplementary Figure 8: Summary prevalence of short birth interval among studies an interval of < 12 months to classify shorth birth interval

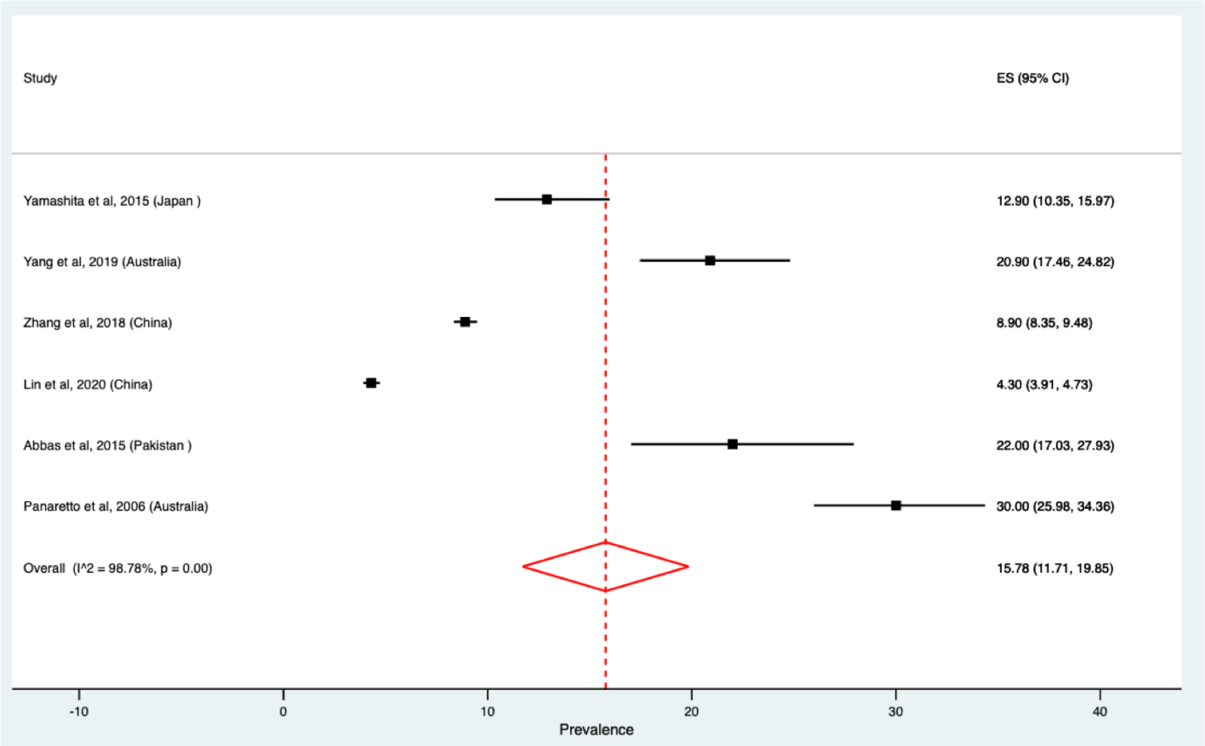

Supplementary Figure 9: Summary prevalence of short birth interval among studies an interval of < 12 months to classify shorth birth interval

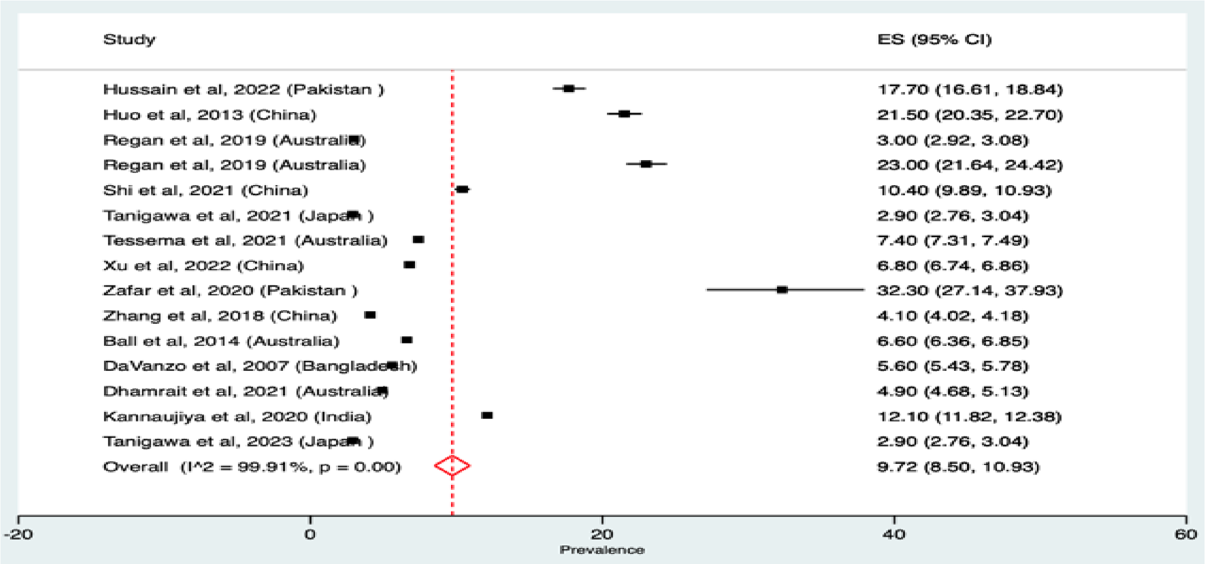

Supplementary Figure 10: Summary prevalence of short birth interval among studies an interval of < 6months to classify shorth birth interval

Summary prevalence by selected countries

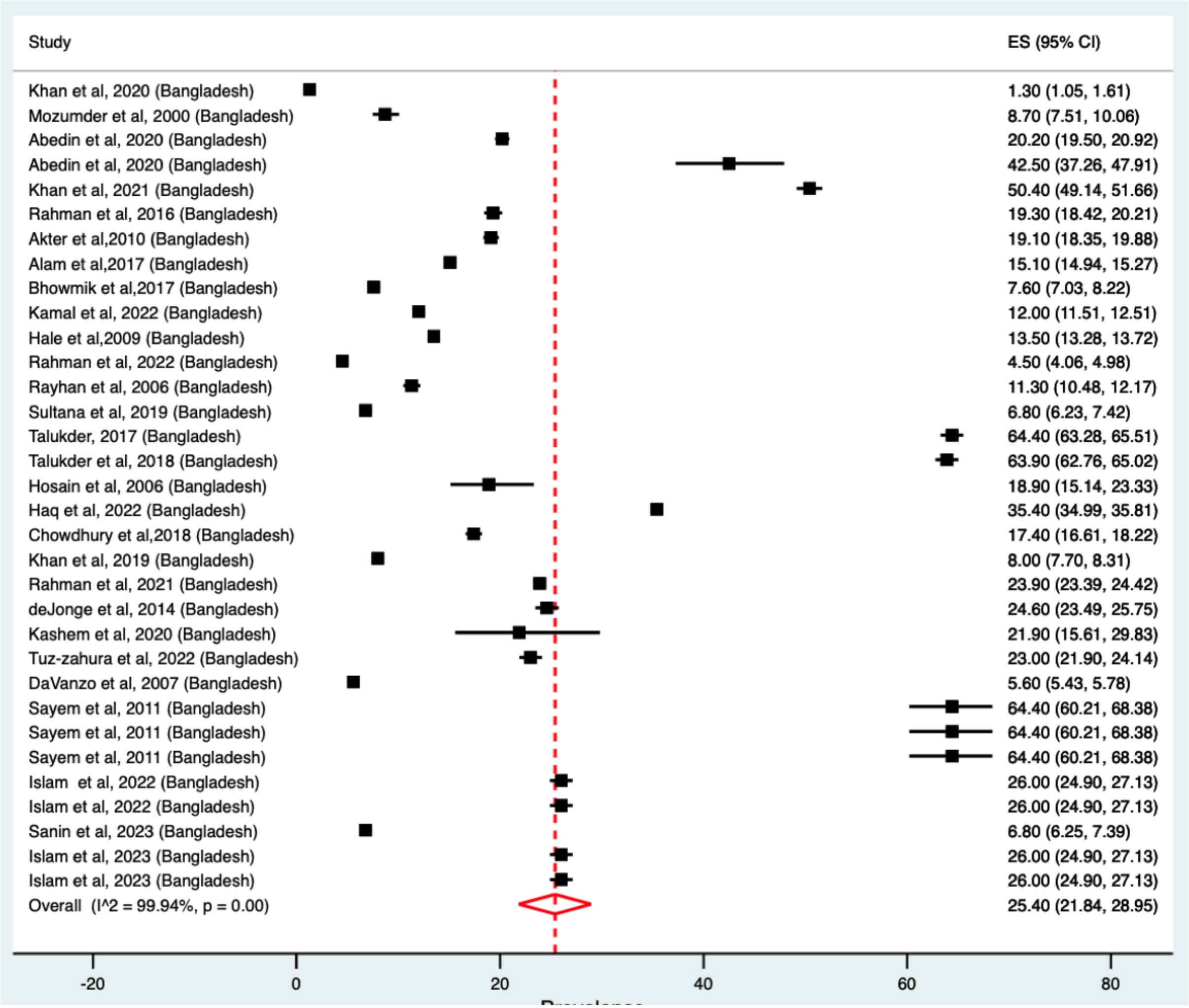

Supplementary Figure 11: Summary prevalence of short birth interval in Bangladesh

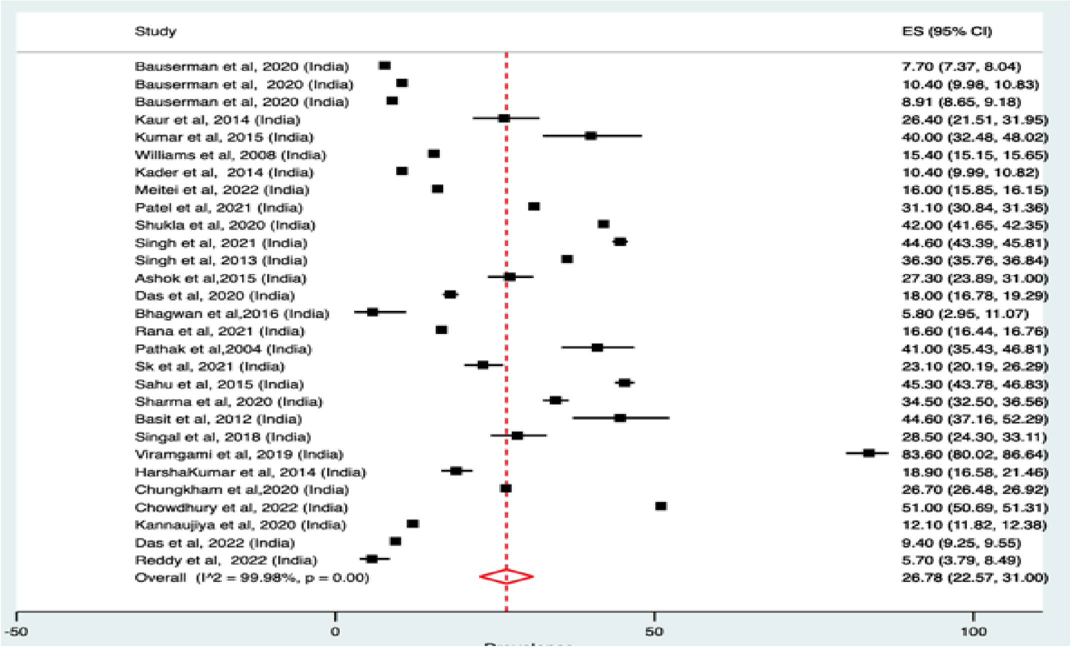

Supplementary Figure 12: Summary prevalence of short birth interval in India

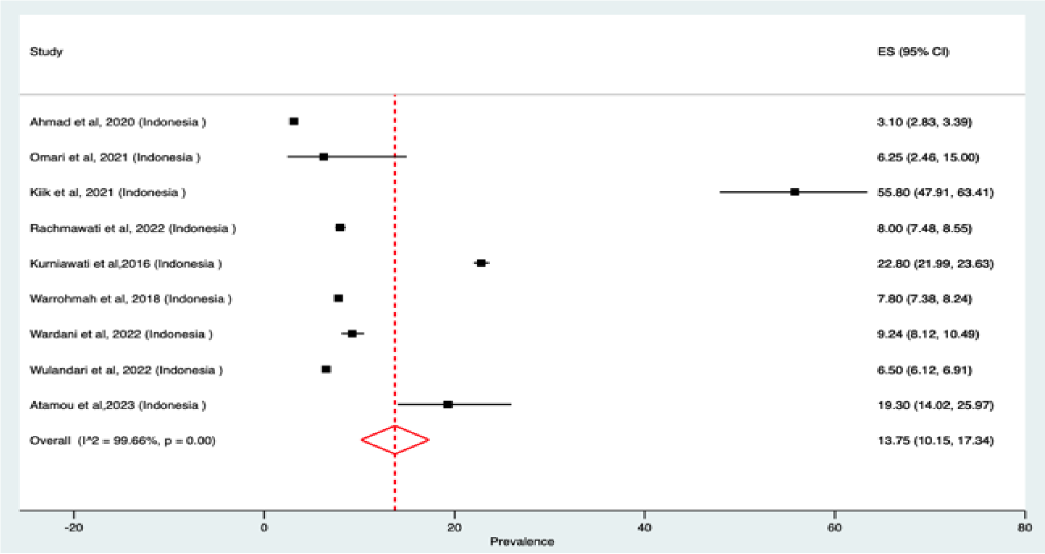

Supplementary Figure 13: Summary prevalence of short birth interval in Indonesia

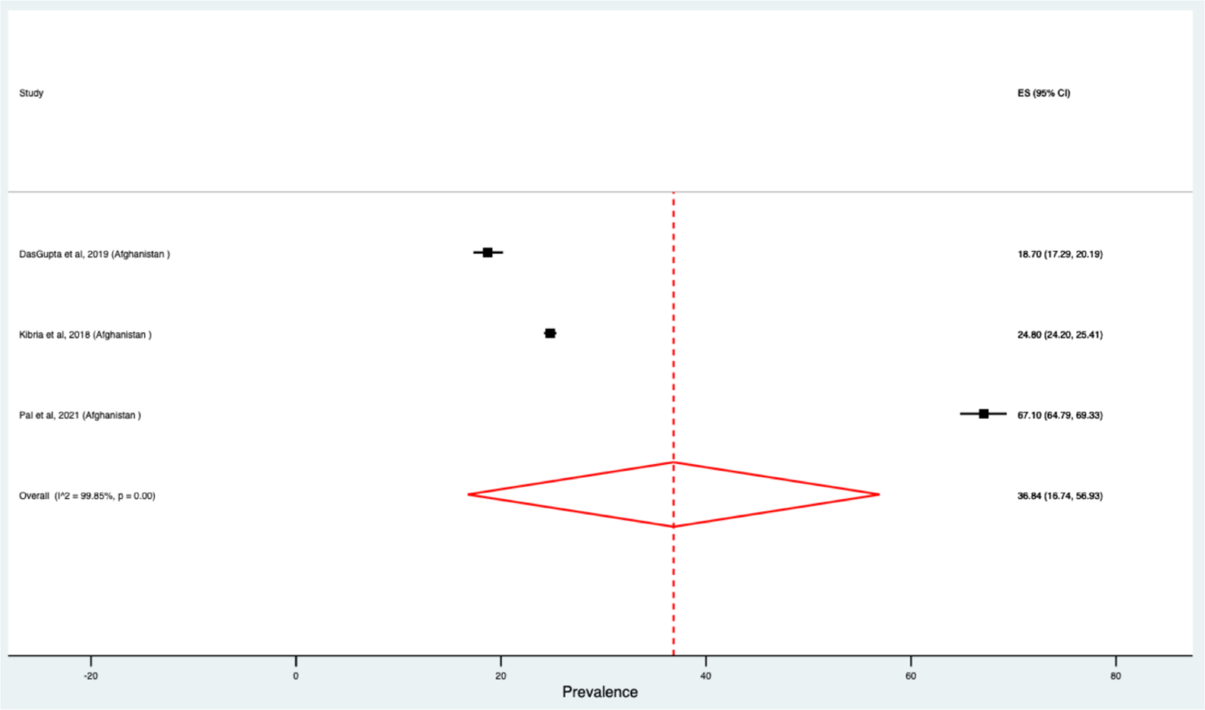

Supplementary Figure 13: Summary prevalence of short birth interval in Afghanistan

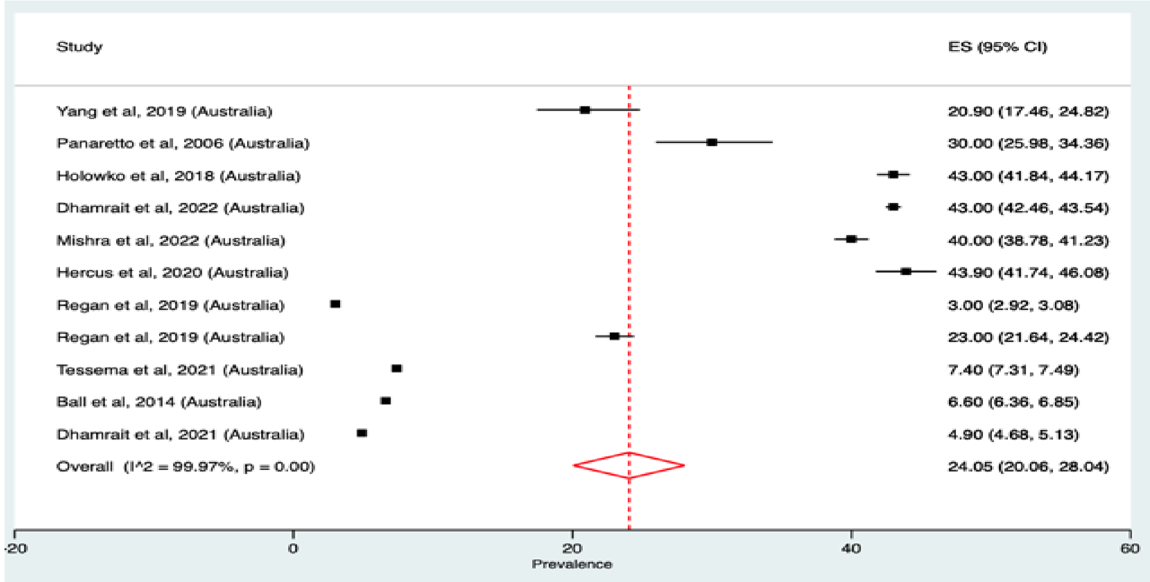

Supplementary Figure 14: Summary prevalence of short birth interval in Australia

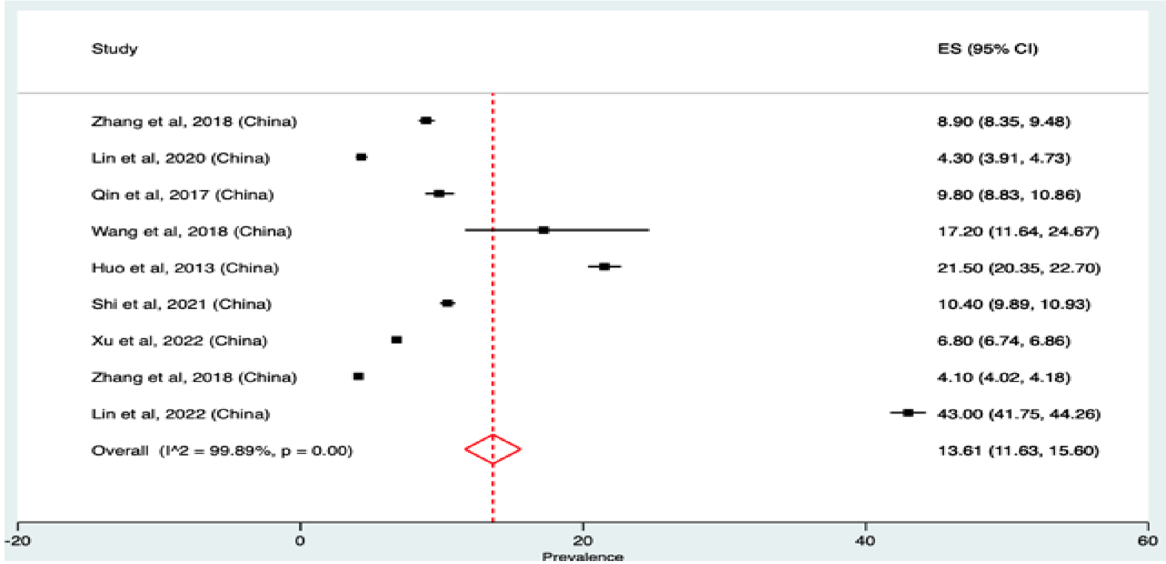

Supplementary Figure 15: Summary prevalence of short birth interval in China

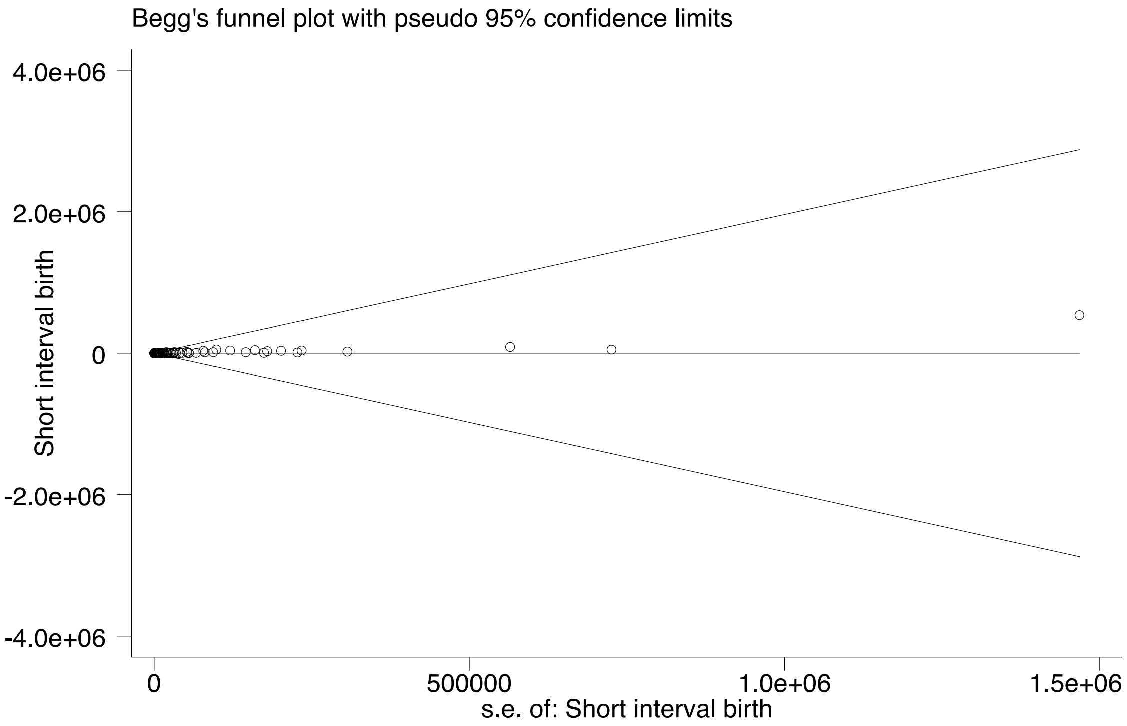

Supplementary figure 16a: Funnel plots showing publication bias regarding the prevalence of short birth interval among studies that followed WHO recommendation.

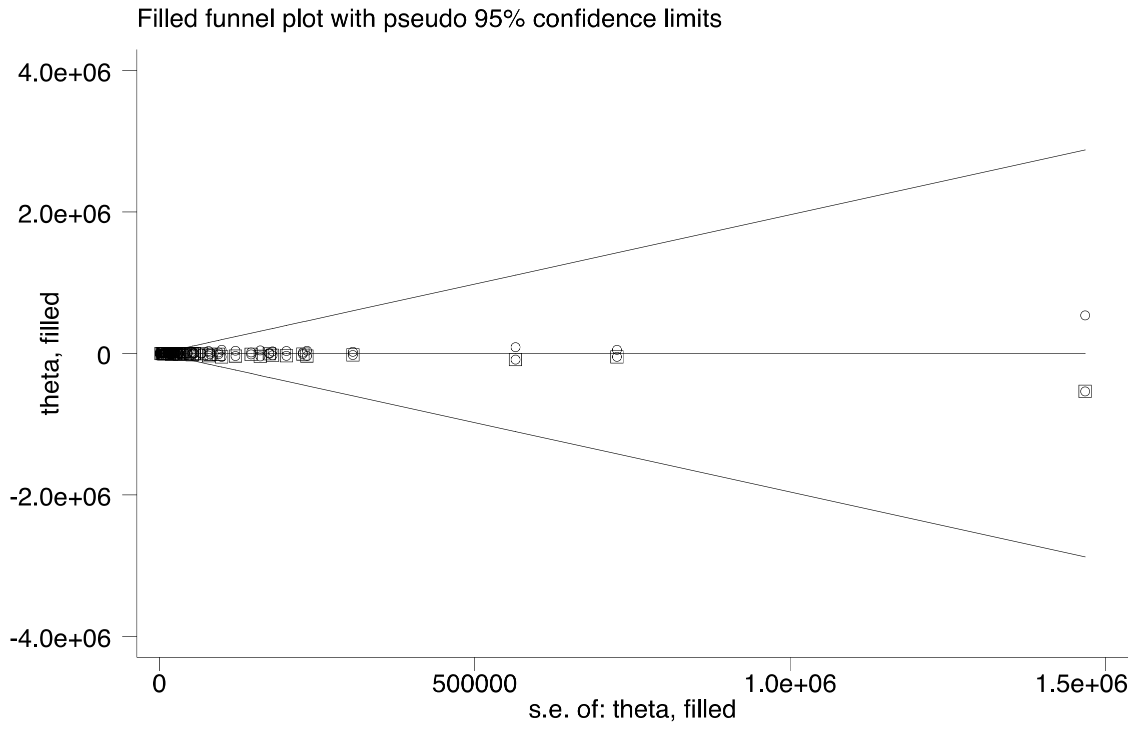

Supplementary figure 16b: Trim and fill estimate addressing evidence of publication bias.

PRISMA 2020 Checklist

Section and Topic	Item #	Checklist item	Location where item is reported
**TITLE**			
Title	1	Identify the report as a systematic review.	Page 1
**ABSTRACT**			
Abstract	2	See the PRISMA 2020 for Abstracts checklist.	Page 2
**INTRODUCTION**			
Rationale	3	Describe the rationale for the review in the context of existing knowledge.	Page 3
Objectives	4	Provide an explicit statement of the objective(s) or question(s) the review addresses.	Page 4
**METHODS**			
Eligibility criteria	5	Specify the inclusion and exclusion criteria for the review and how studies were grouped for the syntheses.	Page 4
Information sources	6	Specify all databases, registers, websites, organisations, reference lists and other sources searched or consulted to identify studies. Specify the date when each source was last searched or consulted.	Page 5
Search strategy	7	Present the full search strategies for all databases, registers and websites, including any filters and limits used.	Page 5 and Supplementary TableS1-5
Selection process	8	Specify the methods used to decide whether a study met the inclusion criteria of the review, including how many reviewers screened each record and each report retrieved, whether they worked independently, and if applicable, details of automation tools used in the process.	Page 6
Data collection process	9	Specify the methods used to collect data from reports, including how many reviewers collected data from each report, whether they worked independently, any processes for obtaining or confirming data from study investigators, and if applicable, details of automation tools used in the process.	Page 6
Data items	10a	List and define all outcomes for which data were sought. Specify whether all results that were compatible with each outcome domain in each study were sought (e.g. for all measures, time points, analyses), and if not, the methods used to decide which results to collect.	Page 6
	10b	List and define all other variables for which data were sought (e.g. participant and intervention characteristics, funding sources). Describe any assumptions made about any missing or unclear information.	Page 6
Study risk of bias assessment	11	Specify the methods used to assess risk of bias in the included studies, including details of the tool(s) used, how many reviewers assessed each study and whether they worked independently, and if applicable, details of automation tools used in the process.	Page 6 and 7

Section and Topic	Item #	Checklist item	Location where item is reported
Effect measures	12	Specify for each outcome the effect measure(s) (e.g. risk ratio, mean difference) used in the synthesis or presentation of results.	Page 6
Synthesis methods	13a	Describe the processes used to decide which studies were eligible for each synthesis (e.g. tabulating the study intervention characteristics and comparing against the planned groups for each synthesis (item #5)).	Page 7
	13b	Describe any methods required to prepare the data for presentation or synthesis, such as handling of missing summary statistics, or data conversions.	Page 7
	13c	Describe any methods used to tabulate or visually display results of individual studies and syntheses.	Page 7
	13d	Describe any methods used to synthesize results and provide a rationale for the choice(s). If meta-analysis was performed, describe the model(s), method(s) to identify the presence and extent of statistical heterogeneity, and software package(s) used.	Page 7
	13e	Describe any methods used to explore possible causes of heterogeneity among study results (e.g. subgroup analysis, meta-regression).	Page 7
	13f	Describe any sensitivity analyses conducted to assess robustness of the synthesized results.	Page 7
Reporting bias assessment	14	Describe any methods used to assess risk of bias due to missing results in a synthesis (arising from reporting biases).	Page 7
Certainty assessment	15	Describe any methods used to assess certainty (or confidence) in the body of evidence for an outcome.	NA
**RESULTS**			
Study selection	16a	Describe the results of the search and selection process, from the number of records identified in the search to the number of studies included in the review, ideally using a flow diagram.	Page 7
	16b	Cite studies that might appear to meet the inclusion criteria, but which were excluded, and explain why they were excluded.	Page 7
Study characteristics	17	Cite each included study and present its characteristics.	Page 8 and supplementary TableS6
Risk of bias in studies	18	Present assessments of risk of bias for each included study.	Page 8 and supplementary TableS7-9
Results of individual studies	19	For all outcomes, present, for each study: (a) summary statistics for each group (where appropriate) and (b) an effect estimate and its precision (e.g. confidence/credible interval), ideally using structured tables or plots.	Page 11
Results of syntheses	20a	For each synthesis, briefly summarise the characteristics and risk of bias among contributing studies.	Page 8
	20b	Present results of all statistical syntheses conducted. If meta-analysis was done, present for each the summary estimate and its precision (e.g. confidence/credible interval) and measures of statistical heterogeneity. If comparing groups, describe the direction of the effect.	Page 11

Section and Topic	Item #	Checklist item	Location where item is reported
	20c	Present results of all investigations of possible causes of heterogeneity among study results.	Page 10
	20d	Present results of all sensitivity analyses conducted to assess the robustness of the synthesized results.	Page 10 and supplementary figure16
Reporting biases	21	Present assessments of risk of bias due to missing results (arising from reporting biases) for each synthesis assessed.	Page 10
Certainty of evidence	22	Present assessments of certainty (or confidence) in the body of evidence for each outcome assessed.	NA
**DISCUSSION**			
Discussion	23a	Provide a general interpretation of the results in the context of other evidence.	Page 13
	23b	Discuss any limitations of the evidence included in the review.	Page 13
	23c	Discuss any limitations of the review processes used.	Page 16
	23d	Discuss implications of the results for practice, policy, and future research.	Page 16
**OTHER INFORMATION**			
Registration and protocol	24a	Provide registration information for the review, including register name and registration number, or state that the review was not registered.	PROSPERO(CRD42023426975)
	24b	Indicate where the review protocol can be accessed, or state that a protocol was not prepared.	Published in BMJ open: https://bmjopen.bmj.com/content/13/12/e076908
	24c	Describe and explain any amendments to information provided at registration or in the protocol.	NA
Support	25	Describe sources of financial or non-financial support for the review, and the role of the funders or sponsors in the review.	None
Competing interests	26	Declare any competing interests of review authors.	None
Availability of data, code and other materials	27	Report which of the following are publicly available and where they can be found: template data collection forms; data extracted from included studies; data used for all analyses; analytic code; any other materials used in the review.	All included in the manuscript

Reference

1 Panaretto K, Lee H, Mitchell M, Larkins S, Manessis V, Buettner P, et al. Risk factors for preterm, low birth weight and small for gestational age birth in urban Aboriginal and Torres Strait Islander women in Townsville. Aust N Z J Public Health. 2006;30:163-70.

2 Yamashita M, Hayashi S, Endo M, Okuno K, Fukui O, Mimura K, et al. Incidence and risk factors for recurrent spontaneous preterm birth: A retrospective cohort study in Japan. Journal of Obstetrics & Gynaecology Research. 2015;41:1708-14.

3 Yang JM, Cheney K, Taylor R, Black K. Interpregnancy intervals and women's knowledge of the ideal timing between birth and conception. BMJ sex. 2019;11:11.

4 Zhang Q, Dang SN, Bai RH, Mi BB, Wang LL, Yan H. Association between maternal interpregnancy interval after live birth or pregnancy termination and birth weight: a quantile regression analysis. SCIENTIFIC REPORTS. 2018;8.

5 Lin J, Liu H, Wu DD, Hu HT, Wang HH, Zhou CL, et al. Long interpregnancy interval and adverse perinatal outcomes: A retrospective cohort study. SCIENCE CHINA-LIFE SCIENCES. 2020;63:898-904.

6 Negi KS, Kandpal SD, Kukreti M. Epidemiological factors affecting low birth weight. JK Science. 2006;8:31-4.

7 Abbasi SRS, Akram MB, Raza H. Maternal demographic determinants of low birth weight babies in District Jhang (Pakistan). Mediterranean Journal of Social Sciences. 2015;6:498-503.

8 Khan JR, Gulshan J. Heterogeneous effects of factors on child nutritional status in Bangladesh using linear quantile mixed model. Biostatistics and Epidemiology. 2020;4:265-81.

9 Mozumder AB, Barkat EK, Kane TT, Levin A, Ahmed S. The effect of birth interval on malnutrition in Bangladeshi infants and young children. J Biosoc Sci. 2000;32:289-300.

10 Abedin S, Arunachalam D. Maternal autonomy and high-risk pregnancy in Bangladesh: the mediating influences of childbearing practices and antenatal care. BMC Pregnancy Childbirth. 2020;20:555.

11 Abedin S, Arunachalam D. Determinants of high-risk childbearing and high-risk pregnancy in Bangladesh. Springer Singapore; 2020. p. 91-104.

12 Ahmad I, Dewi YR. Birth intervals and infant mortality in indonesia. Population Review. 2020;59:73-84.

13 Holowko N, Jones M, Tooth L, Koupil I, Mishra GD. Socioeconomic Position and Reproduction: Findings from the Australian Longitudinal Study on Women's Health. Matern Child Health J. 2018;22:1713-24.
14 Dhamrait G, O'Donnell M, Christian H, Pereira G. Is early childhood development impeded by the birth timing of the younger sibling? PLoS ONE. 2022;17:e0268325.
15 Nakamura Y, Tsuda H, Masahashi Y, Nakamura T, Suzuki M, Fukuhara N, et al. Impact of the interpregnancy interval after cesarean delivery on subsequent perinatal risks: a retrospective study. Arch Gynecol Obstet. 2022:7.
16 Mishra GD, Baneshi MR, Dobson AJ, Tooth LR. Maternal factors associated with interbirth intervals in Australia: Results from a population-based longitudinal study. Birth. 2022;31:31.
17 Kaur S, Upadhyay AK, Srivastava DK, Srivastava R, Pandey ON. Maternal correlates of birth weight of newborn: A hospital based study. Indian Journal of Community Health. 2014;26:187-91.
18 Kluckow H, Panisi L, Larui J, Jatobatu A, Kim D, Hodges L, et al. Socio-demographic predictors of unintended pregnancy and late antenatal booking in Honiara, Solomon Islands. Aust N Z J Obstet Gynaecol. 2018;58:349-57.
19 Kumar V, Sunderam S, Haider S, Kashyap V. A study on status of anaemia in pregnant women attending urban health training centre, RIMS, Ranchi. Indian Journal of Community Health. 2015;26:112-7.
20 Williams EK, Hossain MB, Sharma RK, Kumar V, Pandey CM, Baqui AH. Birth interval and risk of stillbirth or neonatal death: findings from rural north India. J Trop Pediatr. 2008;54:321-7.
21 Kader M, Perera NKPP. Socio-economic and nutritional determinants of low birth weight in India. N A J Med Sci (Hamilt). 2014;6:302-8.
22 Hazel EA, Mohan D, Zeger S, Mullany LC, Tielsch JM, Khatry SK, et al. Demographic, socio-economic, obstetric, and behavioral factors associated with small-and large-for-gestational-age from a prospective, population-based pregnancy cohort in rural Nepal: a secondary data analysis. BMC Pregnancy Childbirth. 2022;22:652.
23 Lin J, Zhou Y, Gu W. The synergistic effects of short inter-pregnancy interval and micronutrients deficiency on third-trimester depression. Front Nutr. 2022;9:12.
24 Khan JR, Tomal JH, Raheem E. Model and variable selection using machine learning methods with applications to childhood stunting in Bangladesh. Inform Health Soc Care. 2021;46:425-42.
25 Naz L, Patel KK, Dilanchiev A. Are socioeconomic status and type of residence critical risk factors of under-five mortality in Pakistan? Evidence from nationally representative survey. Clinical Epidemiology and Global Health. 2021;10.
26 Rahman MS, Howlader T, Masud MS, Rahman ML. Association of Low-Birth Weight with Malnutrition in Children under Five Years in Bangladesh: Do Mother's Education, Socio-Economic Status, and Birth Interval Matter? PLoS ONE. 2016;11:e0157814.
27 Singh R, Mahat S, Singh S, Nyamasege CK, Wagatsuma Y. The relationship between pelvic organ prolapse and short birth intervals in a rural area of Nepal. Trop. 2021;49:5.
28 Wali N, Agho KE, Renzaho AMN. Wasting and associated factors among children under 5 years in five south asian countries (2014–2018): Analysis of demographic health surveys. International Journal of Environmental Research and Public Health. 2021;18.

29 Abeywickrama G, Anuranga C. A decomposition analysis of inequalities in Low birth weight in Sri Lanka: findings from the Demographic and Health survey- 2016. Ceylon Med J. 2020;65:15-22.
30 Alam N, Van Ginneken JK, Bosch AM. Infant mortality among twins and triplets in rural Bangladesh in 1975-2002. Trop Med Int Health. 2007;12:1506-14.
31 Amir-ud-Din R, Naz L, Rubi A, Usman M, Ghimire U. Impact of high-risk fertility behaviours on underfive mortality in Asia and Africa: evidence from Demographic and Health Surveys. BMC PREGNANCY AND CHILDBIRTH. 2021;21.
32 Bhowmik KR, Das S. On exploring and ranking risk factors of child malnutrition in Bangladesh using multiple classification analysis. BMC Nutr. 2017;3:73.
33 Habib MA, Raynes-Greenow C, Soofi SB, Ali N, Nausheen S, Ahmed I, et al. Prevalence and determinants of iron deficiency anemia among non-pregnant women of reproductive age in Pakistan. Asia Pac J Clin Nutr. 2018;27:195-203.
34 Akter S, Rahman JAMS, Rahman MM, Abedin S. The influence of birth spacing on child survival in Bangladesh: a life table approach. World Health Popul. 2010;12:42-56.
35 Kamal SMM, Moniruzzaman M. Birth Interval and its Association With Adverse Childhood Nutritional Outcomes Among Under-Five Children in Bangladesh: A Longitudinal Study. Journal of Nepal Paediatric Society. 2022;41:327-35.
36 Hale L, DaVanzo J, Razzaque A, Rahman M. Which factors explain the decline in infant and child mortality in Matlab, Bangladesh? JOURNAL OF POPULATION RESEARCH. 2009;26:3-20.
37 Murtaza K, Saleem Z, Jabeen S, Alzahrani AK, Kizilbash N, Soofi SB, et al. Impact of interpregnancy intervals on perinatal and neonatal outcomes in a multiethnic Pakistani population. J Trop Pediatr. 2022;68:06.
38 Ismail TAT, Hamzah TNT, Hassan MHM, Mahmood NMZ. Prevalence and factors associated with short birth spacing among Malay women in Kota Bharu, Kelantan, Malaysia. International Medical Journal. 2008;15:131-6.
39 Wardani Y, Huang YL, Chuang YC. Factors Associated with Infant Deaths in Indonesia: An Analysis of the 2012 and 2017 Indonesia Demographic and Health Surveys. J Trop Pediatr. 2022;68:14.
40 Qin C, Mi C, Xia A, Chen W-T, Chen C, Li Y, et al. A first look at the effects of long inter-pregnancy interval and advanced maternal age on perinatal outcomes: A retrospective cohort study. Birth. 2017;44:230-7.
41 Mardiana O, Afiah MZN, Norliza A. Role of sociodemographic, obstetric history and planning of pregnancy in predicting short interpregnancy interval among antenatal mothers in Klang, Selangor. Medical Journal of Malaysia. 2019;74:151-9.
42 Kibria GMA, Burrowes V, Choudhury A, Sharmeen A, Ghosh S, Mahmud A, et al. Determinants of early neonatal mortality in Afghanistan: an analysis of the Demographic and Health Survey 2015. Global health. 2018;14:47.
43 Das Gupta R, Swasey K, Burrowes V, Hashan MR, Al Kibria GM. Factors associated with low birth weight in Afghanistan: a cross-sectional analysis of the demographic and health survey 2015. BMJ Open. 2019;9:e025715.
44 Omari DF, Yusrawati, Yenny SW. Maternal deaths due to obstetric hemorrhage in Padang, Indonesia: A case-control study. Indonesian Journal of Obstetrics and Gynecology. 2021;9:65-9.
45 Kiik SM, Nuwa MS. Maternal factors in stunting among vulnerable children. Jurnal Keperawatan Indonesia. 2021;24:82-9.

46 Meitei WB, Singh A, Ladusingh L. The effects of community clustering on under-five mortality in India: a parametric shared frailty modelling approach. Genus. 2022;78.
47 Myo T, Hong SA, Thepthien B-O, Hongkrailert N. Prevalence and Factors Associated with Postpartum Depression in Primary Healthcare Centres in Yangon, Myanmar. Malays. 2021;28:71-86.
48 Pal SK, Vijay J, Patel KK. Prevalence of under-5 mortality and its associated risk factors in Afghanistan. Children & Youth Services Review. 2021;120:N.PAG-N.PAG.
49 Patel KK, Vijay J, Mangal A, Mangal DK, Gupta SD. Burden of anaemia among children aged 6–59 months and its associated risk factors in India – Are there gender differences? Children & Youth Services Review. 2021;122:N.PAG-N.PAG.
50 Pravana NK, Piryani S, Chaurasiya SP, Kawan R, Thapa RK, Shrestha S. Determinants of severe acute malnutrition among children under 5 years of age in Nepal: a community-based case-control study. BMJ Open. 2017;7:e017084.
51 Rachmawati PD, Kurnia ID, Asih MN, Kurniawati TW, Krisnana I, Arief YS, et al. Determinants of under-five mortality in Indonesia: A nationwide study. J Pediatr Nurs. 2022;65:e43-e8.
52 Rahman MS, Rahman MA, Afroze L, Khan AG, Mahmud ZA, Islam SMS. Determinants of mortality in children aged under two years in Bangladesh using two approaches: an analysis of the Bangladesh Demographic and Health Survey 2014 data. Bangladesh Journal of Medical Science. 2022;21:413-21.
53 Rayhan MI, Khan MSH. Factors causing malnutrition among under five children in Bangladesh. Pakistan Journal of Nutrition. 2006;5:558-62.
54 Sanjel K, Onta SR, Amatya A, Basel P. Patterns and determinants of essential neonatal care utilization among underprivileged ethnic groups in Midwest Nepal: a mixed method study. BMC Pregnancy Childbirth. 2019;19:310.
55 Shakya K, McMurray C. Neonatal mortality and maternal health care in Nepal: searching for patterns of association. J Biosoc Sci. 2001;33:87-105.
56 Shukla A, Kumar A, Mozumdar A, Aruldas K, Acharya R, Ram F, et al. Association between modern contraceptive use and child mortality in India: A calendar data analysis of the National Family Health Survey (2015-16). SSM Popul Health. 2020;11:100588.
57 Singh BP, Madhusudan JV, Singh S. Mother's education and mortality under age three: An investigation in presence of some socio-demographic correlates. International Journal of Current Research and Review. 2021;13:20-8.
58 Singh R, Tripathi V. Maternal factors contributing to under-five mortality at birth order 1 to 5 in India: a comprehensive multivariate study. Springerplus. 2013;2:284.
59 Sultana P, Rahman MM, Akter J. Correlates of stunting among under-five children in Bangladesh: a multilevel approach. BMC Nutr. 2019;5:41.
60 Talukder A. Factors Associated with Malnutrition among Under-Five Children: Illustration using Bangladesh Demographic and Health Survey, 2014 Data. Children (Basel). 2017;4:19.
61 Talukder A, Razu SR, Hossain MZ. Factors affecting stunting among children under five years of age in Bangladesh. Family Medicine & Primary Care Review. 2018;20:356-62.
62 Warrohmah ANI, Berliana SM, Nursalam N, Efendi F, Haryanto J, Has EMM, et al., editors. Analysis of the Survival of Children under Five in Indonesia and Associated Factors2018: Institute of Physics Publishing.

63 Ashok NC, Santosh Kumar A, Koppad R, Sunil Kumar D, Dhar M, Chandrashekar SV. PEM in relation to birth order and birth interval in children aged 1-6 years in urban slums of Mysore city. Indian Journal of Public Health Research and Development. 2015;6:308-13.
64 Dahal K, Yadav DK, Baral D, Yadav BK. Determinants of severe acute malnutrition among under 5 children in Satar community of Jhapa, Nepal. PLoS ONE. 2021;16:e0245151.
65 Das S, Chanani S, Shah More N, Osrin D, Pantvaidya S, Jayaraman A. Determinants of stunting among children under 2 years in urban informal settlements in Mumbai, India: evidence from a household census. J Health Popul Nutr. 2020;39:10.
66 Farid-ul-Hasnain S, Sophie R. Prevalence and risk factors for stunting among children under 5 years: a community based study from Jhangara town, Dadu Sindh. JPMA J Pak Med Assoc. 2010;60:41-4.
67 Hakim N, Lakhan H, Shah FJ, Shams ul H, Muntaqa M, Masood M. Frequency of Maternal Factors in Patients of Stillbirth in Pakhtoon Families Visiting Public Hospitals of Peshawar. PAKISTAN JOURNAL OF MEDICAL & HEALTH SCIENCES. 2021;15:2971-3.
68 Helova A, Hearld KR, Budhwani H. Associates of Neonatal, Infant and Child Mortality in the Islamic Republic of Pakistan: A Multilevel Analysis Using the 2012-2013 Demographic and Health Surveys. Matern Child Health J. 2017;21:367-75.
69 Hosain GM, Chatterjee N, Begum A, Saha SC. Factors associated with low birthweight in rural Bangladesh. J Trop Pediatr. 2006;52:87-91.
70 Chungkham HS, Sahoo H, Marbaniang SP. Birth interval and childhood undernutrition: Evidence from a large scale survey in India. Clinical Epidemiology and Global Health. 2020;8:1189-94.
71 Sanin KI, Khanam M, Rita RS, Haque MA, Ahmed T. Common factors influencing childhood undernutrition and their comparison between Sylhet, the most vulnerable region, and other parts of Bangladesh: Evidence from BDHS 2007–18 rounds. Front Nutr. 2023;9.
72 Das M, Jana A, Muhammad T. Understanding the associations between maternal high-risk fertility behaviour and child nutrition levels in India: evidence from the National Family Health Survey 2015-2016. Sci. 2022;12:17742.
73 Wulandari F, Mahmudiono T, Rifqi MA, Helmyati S, Dewi M, Yuniar CT. Maternal Characteristics and Socio-Economic Factors as Determinants of Low Birth Weight in Indonesia: Analysis of 2017 Indonesian Demographic and Health Survey (IDHS). Int J Environ Res Public Health. 2022;19:26.
74 Acharya D, Gautam S, Poder TG, Lewin A, Gaussen A, Lee K, et al. Maternal and dietary behavior-related factors associated with preterm birth in Southeastern Terai, Nepal: A cross sectional study. Front. 2022;10:946657.
75 Wang N, Lu W, Xu Y, Mao S, He M, Lin X, et al. Recurrence of diet-treated gestational diabetes in primiparous women in northern Zhejiang, China: Epidemiology, risk factors and implications. J Obstet Gynaecol Res. 2018;44:1391-6.
76 Bhagwan D, Kumar A, Rao CR, Kamath A. Prevalence of Anaemia among Postnatal Mothers in Coastal Karnataka. J Clin Diagn Res. 2016;10:LC17-20.
77 Rana S, Panza A. PREVALENCE AND FACTORS ASSOCIATED WITH UNDERNUTRITION AMONG CHILDREN AGED 0-59 MONTHS IN MUGU DISTRICT, NEPAL. JOURNAL OF HEALTH RESEARCH. 2015;29:S117-S24.
78 Angeles-Agdeppa I, Gayya-Amita PI, Capanzana MV. Drivers of Stunting Among 0-23 Months Old Filipino Children Included in the 2003 and 2011 National Nutrition Survey. INTERNATIONAL JOURNAL OF CHILD HEALTH AND NUTRITION. 2019;8:74-85.
79 Rana MJ, Cleland J, Sekher TV, Padmadas SS. Disentangling the effects of reproductive behaviours and fertility preferences on child growth in India. Popul Stud (Camb). 2021;75:37-50.

80 Haq I, Alam M, Islam A, Rahman M, Latif A, Methun MIH, et al. Influence of sociodemographic factors on child mortality in Bangladesh: a multivariate analysis. Journal of Public Health (09431853). 2022;30:1079-86.
81 Pathak P, Kapil U, Kapoor SK, Saxena R, Kumar A, Gupta N, et al. Prevalence of multiple micronutrient deficiencies amongst pregnant women in a rural area of Haryana. Indian J Pediatr. 2004;71:1007-14.
82 Asif MF, Meherali S, Abid G, Khan MS, Lassi ZS. Predictors of Child's Health in Pakistan and the Moderating Role of Birth Spacing. Int J Environ Res Public Health. 2022;19:03.
83 Chowdhury M, Dibley MJ, Alam A, Huda TM, Raynes-Greenow C. Household Food Security and Birth Size of Infants: Analysis of the Bangladesh Demographic and Health Survey 2011. Curr. 2018;2:nzy003.
84 Nausheen S, Bhura M, Hackett K, Hussain I, Shaikh Z, Rizvi A, et al. Determinants of short birth intervals among married women: a cross-sectional study in Karachi, Pakistan. BMJ Open. 2021;11:e043786.
85 Chowdhury S, Singh A, Kasemi N, Chakrabarty M, Singh S. Short birth interval and associated factors in rural India: A cross-sectional study. J Biosoc Sci. 2022:1-20.
86 de Jonge HCC, Azad K, Seward N, Kuddus A, Shaha S, Beard J, et al. Determinants and consequences of short birth interval in rural Bangladesh: a cross-sectional study. BMC Pregnancy Childbirth. 2014;14:427.
87 Islam MZ, Rahman M, Khan N. Exploring the association between child nutritional disorders and short birth interval: Evidence from 2017/18 Bangladesh Demographic and Health Survey data. Clin Epidemiol Global Health. 2023;20.
88 Islam MZ, Rahman MM, Khan MN. Effects of short birth interval on different forms of child mortality in Bangladesh: Application of propensity score matching technique with inverse probability of treatment weighting. PLoS ONE. 2023;18:e0284776.
89 Asif MF, Ishtiaq S, Abbasi NI, Tahir I, Abid G, Lassi ZS. The Interaction Effect of Birth Spacing and Maternal Healthcare Services on Child Mortality in Pakistan. Children (Basel). 2023;10:12.
90 Kashem SR, Arokiasamy JT, Khanam ST, Anthonisamy AN, Bhatt P, Islam M. Sociodemographic factors associated with birth interval: a study among rural women in Chandina, Comilla, Bangladesh. Journal of Public Health (09431853). 2020;28:11-5.
91 Tuz-Zahura F, Sen KK, Nilima S, Bari W. Can women's 3E index impede short birth interval? evidence from Bangladesh Demographic and Health Survey, 2017-18. PLoS ONE. 2022;17:e0263003.
92 Sk R, Banerjee A, Rana MJ. Nutritional status and concomitant factors of stunting among pre-school children in Malda, India: A micro-level study using a multilevel approach. BMC Public Health. 2021;21:1690.
93 Thakur J, Bhatta NK, Poudel P, Shah GS, Singh RR. Assessment of nutritional status of children attending paediatrics OPD of a tertiary care hospital in eastern nepal. Journal of Nepal Paediatric Society. 2018;37:209-12.
94 Atamou L, Rahmadiyah DC, Hassan H, Setiawan A. Analysis of the Determinants of Stunting among Children Aged below Five Years in Stunting Locus Villages in Indonesia. Healthcare (Basel). 2023;11:09.
95 Huo X-X, Gao E-S, Cheng Y-M, Luo L, Liang H, Huang G-Y, et al. Effect of interpregnancy interval after a mifepristone-induced abortion on neonatal outcomes in subsequent pregnancy. Contraception. 2013;87:38-44.

96 Marinovich ML, Regan AK, Gissler M, et al. Associations between interpregnancy interval and preterm birth by previous preterm birth status in four high-income countries: a cohort study. BJOG: An International Journal of Obstetrics and Gynaecology. 2021;128:1134-43.
97 Regan AK, Ball SJ, Warren JL, Malacova E, Padula A, Marston C, et al. A Population-Based Matched-Sibling Analysis Estimating the Associations Between First Interpregnancy Interval and Birth Outcomes. Am J Epidemiol. 2019;188:9-16.
98 Regan AK, Gissler M, Magnus MC, Haberg SE, Ball S, Malacova E, et al. Association between interpregnancy interval and adverse birth outcomes in women with a previous stillbirth: an international cohort study. Lancet. 2019;393:1527-35.
99 Shi G, Zhang B, Kang Y, Dang S, Yan H. Association of Short and Long Interpregnancy Intervals with Adverse Birth Outcomes: Evidence from a Cross-Sectional Study in Northwest China. Int J Gen Med. 2021;14:2871-81.
100 Tanigawa K, Ikehara S, Cui M, Kawanishi Y, Kimura T, Ueda K, et al. Association between interpregnancy interval and risk of preterm birth and its modification by folate intake: the Japan Environment and Children's Study. J Epidemiol. 2021;22:22.
101 Tessema GA, Marinovich ML, Håberg SE, Gissler M, Mayo JA, Nassar N, et al. Interpregnancy intervals and adverse birth outcomes in high-income countries: An international cohort study. PLoS One. 2021;16:e0255000.
102 Xu T, Miao H, Chen Y, Luo L, Guo P, Zhu Y. Association of Interpregnancy Interval With Adverse Birth Outcomes. JAMA netw. 2022;5:e2216658.
103 Zafar S, Naurin R, Asif HF, Majeed T, Mahmood Z. Short inter-pregnancy interval in multiparous females and impact on preterm delivery. PAKISTAN JOURNAL OF MEDICAL & HEALTH SCIENCES. 2020;14:1128-30.
104 Zhang L, Shen S, He J, Chan F, Lu J, Li W, et al. Effect of Interpregnancy Interval on Adverse Perinatal Outcomes in Southern China: A Retrospective Cohort Study, 2000-2015. Paediatr Perinat Epidemiol. 2018;32:131-40.
105 Arshad A, Javaid MK, Rehman A. Comparison of Perinatal Outcome (Low Birth Weight, Preterm Delivery) in Women with < 6 Month Versus 12-17 Months of Interpregnancy Birth Interval. PAKISTAN JOURNAL OF MEDICAL & HEALTH SCIENCES. 2021;15:2742-5.
106 Ball SJ, Pereira G, Jacoby P, de Klerk N, Stanley FJ. Re-evaluation of link between interpregnancy interval and adverse birth outcomes: retrospective cohort study matching two intervals per mother. Bmj. 2014;349:g4333.
107 DaVanzo J, Hale L, Razzaque A, Rahman M. Effects of interpregnancy interval and outcome of the preceding pregnancy on pregnancy outcomes in Matlab, Bangladesh. Bjog. 2007;114:1079-87.
108 Dhamrait GK, Taylor CL, Pereira G. Interpregnancy intervals and child development at age 5: a population data linkage study. BMJ Open. 2021;11:e045319.
109 Kannaujiya AK, Kumar K, Upadhyay AK, McDougal L, Raj A, Singh A. Short interpregnancy interval and low birth weight births in India: Evidence from National Family Health Survey 2015-16. SSM Popul Health. 2020;12:100700.
110 Tanigawa K, Ikehara S, Cui M, Kawanishi Y, Kimura T, Ueda K, et al. Association Between Interpregnancy Interval and Risk of Preterm Birth and Its Modification by Folate Intake: The Japan Environment and Children's Study. J Epidemiol. 2023;33:113-9.
111 Razzaque A, Da Vanzo J, Rahman M, Gausia K, Hale L, Khan MA, et al. Pregnancy spacing and maternal morbidity in Matlab, Bangladesh. Int J Gynaecol Obstet. 2005;89 Suppl 1:S41-S9.
112 Hussain R. Risk factors for neonatal mortality in low-income population subgroups in Karachi, Pakistan. Community Genet. 2002;5:249-56.

113 Reddy KM, Ravula SR, Palakollu S, Betha K. Prevalence of preterm birth and perinatal outcome: A rural tertiary teaching hospital-based study. J Fam Med Prim Care. 2022;11:3909-14.
114 Ashfaq M, Mateen A, Mateen H, Hanif A. Frequency of Short Interpregnancy Interval in Females with Preterm Birth. PAKISTAN JOURNAL OF MEDICAL & HEALTH SCIENCES. 2017;11:582-4.
115 Khan JR, Biswas RK. Influence of parental education on child mortality in Bangladesh: repeated cross-sectional surveys. Biodemography Soc Biol. 2019;65:214-26.
116 Hercus A, Dekker G, Leemaqz S. Primipaternity and birth interval; independent risk factors for preeclampsia. J Matern-Fetal Neonatal Med. 2020;33:303-6.
117 Sayem AM, Sanaullah Nury ATM. Examination of birth intervals: Marriage to first, first to second and mean birth intervals in Bangladeshi women. Asia-Pacific Social Science Review. 2011;11:1-18.
118 Karkee R, Lee AH. Birth Spacing of Pregnant Women in Nepal: A Community-Based Study. Front. 2016;4:205.
119 Rahman A, Hossain Z, Kabir E, Rois R. Machine Learning Algorithm for Analysing Infant Mortality in Bangladesh. Springer Science and Business Media Deutschland GmbH; 2021. p. 205-19.
120 Sahu D, Nair S, Singh L, Gulati BK, Pandey A. Levels, trends & predictors of infant & child mortality among Scheduled Tribes in rural India. Indian J Med Res. 2015;141:709-19.
121 Sharma J, Pandey S, Negandhi P. Determinants of suboptimal breastfeeding in Haryana - An analysis of national family health survey-4 data. Indian J Public Health. 2020;64:285-94.
122 Basit A, Nair S, Chakraborthy K, Darshan B, Kamath A. Risk factors for under-nutrition among children aged one to five years in Udupi taluk of Karnataka, India: A case control study. Australas Med J. 2012;5:163-7.
123 Kamal A, Shakeel A. Differentials and determinants of neonatal mortality in Pakistan: A cross sectional analysis; Pakistan Demographic and Health Survey (2017-18). JPMA J Pak Med Assoc. 2021;71:900-4.
124 Singal N, Setia G, Taneja BK, Singal KK. Factors associated with maternal anaemia among pregnant women in rural India. Bangladesh Journal of Medical Science. 2018;17:583-92.
125 Islam MZ, Islam MM, Rahman MM, Khan MN. Prevalence and risk factors of short birth interval in Bangladesh: Evidence from the linked data of population and health facility survey. PLOS Global Public Health. 2022;2:e0000288.
126 Islam MA, Khan MNA, Raihan H, Barna SD. Exploring the Influencing Factors for Contraceptive Use among Women: A Meta-Analysis of Demographic and Health Survey Data from 18 Developing Countries. International Journal of Reproductive Medicine. 2022;2022.
127 Viramgami AP, Verma PB, Vala MC, Sharma S. A Cross-Sectional Study to Assess Reproductive and Child Health Profile of Working Women Residing in Urban Slums of Rajkot City. Indian J. 2019;44:313-6.
128 Harsha Kumar H, Gupta S, Ruhela S, Tanya S. A retrospective study on magnitude and factors associated with anemia in postnatal period from coastal South India. ann. 2014;4:775-9.
129 Roberts CL, Algert CS, Ford JB, Nippita TA, Morris JM. Association between interpregnancy interval and the risk of recurrent loss after a midtrimester loss. Hum Reprod. 2016;31:2834-40.

130 Bauserman M, Nowak K, Nolen TL, Patterson J, Lokangaka A, Tshefu A, et al. The relationship between birth intervals and adverse maternal and neonatal outcomes in six low and lower-middle income countries. Reproductive health. 2020;17:1-10.

131 Khan JR, Bari W, Latif AHMM. Trend of determinants of birth interval dynamics in Bangladesh. BMC Public Health. 2016;16:934.

132 Upadhyay UD, Hindin MJ. Do higher status and more autonomous women have longer birth intervals? Results from Cebu, Philippines. Soc Sci Med. 2005;60:2641-55.

133 Mehata S, Paudel YR, Mehta R, Dariang M, Poudel P, Barnett S. Unmet need for family planning in Nepal during the first two years postpartum. Biomed Res Int. 2014;2014:649567.

134 Gebremedhin AT, Regan AK, Ball S, Betran AP, Foo D, Gissler M, et al. Interpregnancy interval and hypertensive disorders of pregnancy: A population-based cohort study. Paediatr Perinat Epidemiol. 2021;35:404-14.

135 Gebremedhin AT, Regan AK, Ball S, Betran AP, Foo D, Gissler M, et al. Effect of interpregnancy interval on gestational diabetes: a retrospective matched cohort study. Ann Epidemiol. 2019;39:33-8.e3.

136 Gebremedhin AT, Tessema GA, Regan AK, Pereira GF. Association between interpregnancy interval and pregnancy complications by history of complications: A population-based cohort study. BMJ Open. 2021;11.

137 Gebremedhin AT, Tessema GA, Regan AK, et al. Association between interpregnancy interval and hypertensive disorders of pregnancy: Effect modification by maternal age. Paediatric and Perinatal Epidemiology. 2021;35:415-24.

138 Murphy M, Wang D. Do previous birth interval and mother's education influence infant survival? A bayesian model averaging analysis of Chinese data. Population Studies. 2001;55:37-47.

139 Shaikh NB, Memon E, Sultana AZ, Shaikh S, Chohan MN. Impact of inter pregnancy interval on outcome among pregnant women with history of miscarriage. RAWAL MEDICAL JOURNAL. 2022;47:97-100.

140 Fatima M, Naz U, Hira AK, Habib A, Kazi PS, Majeed H. Association Between Pre-Term Labour and Inter Pregnancy Interval. PAKISTAN JOURNAL OF MEDICAL & HEALTH SCIENCES. 2021;15:3137-9.
